# Virus replication in the honey bee parasite, *Varroa destructor*

**DOI:** 10.1128/jvi.01149-23

**Published:** 2023-11-15

**Authors:** James E. Damayo, Rebecca C. McKee, Gabriele Buchmann, Amanda M. Norton, Alyson Ashe, Emily J. Remnant

**Affiliations:** 1 School of Life and Environmental Sciences, University of Sydney, Sydney, New South Wales, Australia; 2 Institute of Plant Genetics, Heinrich-Heine University, Duesseldorf, Germany; 3 Academic Support Unit, Research and Advanced Instrumentation, University of the Sunshine Coast, Sippy Downs, Queensland, Australia; Wageningen University & Research, Wageningen, the Netherlands

**Keywords:** honey bee viruses, antiviral siRNA, RNA interference, mite vector, virus replication

## Abstract

The ectoparasitic mite *Varroa destructor* is the leading threat to the health of Western honey bees (*Apis mellifera*) primarily through its action as a vector of viruses. However, it is unclear which of the viruses transmitted by *V. destructor* actively infect and replicate in mites, which could facilitate increased transmission. To better understand the role of *V. destructor* as a vector, we took advantage of differences between bee and mite antiviral RNA interference pathways to identify the host specificity of replicating viruses. We used small RNA sequencing of individual *V. destructor* mites to examine viral small interfering RNA (vsiRNA) profiles of deformed wing virus genotypes (DWV-A and DWV-B), associated with colony declines, as well as nine other viruses present in our samples. We found active replication of six *V. destructor*-associated viruses, including a novel virus, Varroa destructor virus 9 (VDV-9), and replication of two honey bee-associated viruses, including both DWV-A and -B genotypes, confirming that mites are biological vectors for important bee pathogens. We show that the antiviral RNAi response can be used to define the host range of viruses in host-parasite interactions, such as honey bees and their parasites, enabling a better understanding of the role of a vector in the evolution and spread of honey bee pathogens.

**IMPORTANCE:**

The parasitic mite *Varroa destructor* is a significant driver of worldwide colony losses of our most important commercial pollinator, the Western honey bee *Apis mellifera*. Declines in honey bee health are frequently attributed to the viruses that mites vector to honey bees, yet whether mites passively transmit viruses as a mechanical vector or actively participate in viral amplification and facilitate replication of honey bee viruses is debated. Our work investigating the antiviral RNA interference response in *V. destructor* demonstrates that key viruses associated with honey bee declines actively replicate in mites, indicating that they are biological vectors, and the host range of bee-associated viruses extends to their parasites, which could impact virus evolution, pathogenicity, and spread.

## INTRODUCTION

The intimate association between a parasite and host provides ample opportunity for the reciprocal exchange of microbes, such as viruses. Cross-species transmission of viruses is exemplified in arthropod vectors such as mosquitoes, ticks, and aphids, although active viral replication and infection may not always occur in both the vector and host. Some vectors are mechanical, transferring viral particles between hosts via contact without playing an active role in the virus replication cycle, such as many semi-persistent plant-infecting viruses transmitted by aphid vectors (1). Alternatively, biological vectors like *Aedes* mosquitoes play an essential part of the infectious cycle of dengue and yellow fever viruses, where viral replication or maturation occurs within the vector, facilitating infectivity in human hosts (2). Biological vectors may require increased disease tolerance mechanisms to withstand viral infections and maintain fitness while harboring high viral loads as a consequence of viral replication (3).

Western honey bees (*Apis mellifera*) are of critical importance to the agricultural industry as pollinators for a diverse range of horticultural crops (4). Within the last few decades, however, the health of honey bees has declined across most of the globe, exacerbated by the spread of exotic parasites and pathogens, which has been facilitated by the international honey bee trade (5). In particular, the movement of *A. mellifera* into Asia exposed Western honey bees to mites of the genus *Varroa,* obligate ectoparasites originally restricted to the Eastern hive bee, *Apis cerana* (6). In the decades following, two mite species, *Varroa destructor* and *Varroa jacobsoni,* switched hosts from *A. cerana* to *A. mellifera,* with *V. destructor,* in particular, rapidly dispersing across the globe, driving widespread colony losses (7–9).

Mites physiologically affect honey bees by feeding on the fat body tissue of juvenile and adult bees, depleting protein, carbohydrate, and water contents (10, 11). Additionally, *V. destructor* is an efficient vector of viruses, which it can acquire during feeding on infected honey bee hosts, increasing dissemination and dispersal of these viruses within and between colonies (12–14). *Varroa* mites have drastically altered the honey bee viral landscape, as demonstrated during the spread of *V. destructor* throughout New Zealand, where dynamic shifts occurred in the prevalence and load of multiple common honey bee viruses in the years following *V. destructor* introduction (15). Initial increases in Kashmir bee virus, sacbrood virus (SBV), and black queen cell virus (BQCV) were eventually superseded by the deformed wing virus (DWV), which now remains the predominant virus in New Zealand colonies (16). Similarly, in Hawaii, the introduction of *V. destructor* led to an increased prevalence of DWV in managed colonies from ~10% to 100% and an overall reduction in DWV genetic diversity, resulting in the selection of a single dominant DWV genotype (17). The association of DWV with *V. destructor* infestation is frequently implicated as a major driver of colony declines (18, 19), due to an increased abundance of mites leading to higher DWV loads (20), which in turn is linked to weaker colonies (21, 22).

DWV is an icosahedral, positive-sense, single-stranded RNA virus belonging to the *Iflaviridae* family and exists as a multi-strain viral complex (23). Two DWV genetic variants, DWV-A and DWV-B, are closely related with 85% nucleotide identity and are both frequently identified at high abundance in *V. destructor-*infested colonies. An additional two genotypes have been characterized, which are more distantly related; DWV-C (24) and DWV-D [“Egypt bee virus” (25)]; these variants are more restricted in their distribution or historical occurrence. Although DWV-A and -B genotypes are both widespread, they vary in their geographic distribution and their global prevalence has changed over time (26). Historically, DWV-A was the most prevalent genotype; however, over time DWV-B has increased and it is predicted that it will eventually surpass DWV-A (27–29). Frequent coinfections have led to multiple instances of recombination between DWV-A and -B genomes, resulting in the presence of stable recombinant genotypes (30, 31). The different genotypes and recombinants differ in characteristics such as viral accumulation, competition, replication rate, and virulence (27, 32).

The mechanisms by which *Varroa* leads to increased viral prevalence and load are multifaceted. In the absence of *Varroa,* virus transmission between bees normally occurs via the fecal-oral route or the oral transfer of liquids by trophallaxis (33), generally leading to covert infections that rarely present with disease symptoms (13). *Varroa* parasitism introduces an alternate, vector-mediated transmission route, facilitating direct injection of viral particles into the body, leading to systemic, overt infections that circumvent innate defenses provided by the gut and cuticle (34). Increased viral loads and disease symptoms could, therefore, directly result from passive, mechanical vectoring of viral particles via mite mouthparts or gut contents as mites sequentially feed on new hosts (35). However, it is becoming increasingly evident that *V. destructor* is also a biological vector for DWV, with strong evidence of active replication of DWV-B and recombinant DWV genotypes within *Varroa* tissues (36–38). Replication of DWV within *Varroa* changes the evolutionary dynamics of virus transmission, leading to higher viral loads in mites, larger doses inoculated into bees, and selection for genotypes that can replicate in both vector and host, with potentially higher virulence in honey bees (39). There remains some contention regarding whether all DWV genotypes, in particular, DWV-A, are able to replicate in mites (36), with experimental evidence suggesting that DWV-A is transmitted by mites in a non-propagative manner (40).

In addition to DWV, *V. destructor* carries a diverse virome that may contain other honey bee-infecting viruses, along with a suite of *Varroa-*infecting viruses. Like most eukaryotes, *V. destructor-*associated viruses are primarily single-stranded RNA viruses (41). At least 11 positive-sense RNA viruses (+ssRNA) have been identified infecting *V. destructor* (42–46), as well as three negative-sense RNA viruses (−ssRNA; 44, 47), and three DNA viruses (47–49). Some viruses such as Apis rhabdovirus-1 (ARV-1), Apis rhabdovirus-2 (ARV-2), and Varroa destructor virus-2 (VDV-2) are found in virtually all *V. destructor* transcriptomes examined (16, 39).

The obligate association between *V. destructor* mites and their honey bee hosts makes it challenging to definitively identify which viruses are able to replicate in which species. Many studies attempt to identify RNA virus replication using strand-specific RT-PCR to detect the antigenome of replicating single-stranded RNA viruses, which produce double-stranded RNA virus intermediates during virus replication (38, 50). While this method may be suitable in non-parasitic organisms, parasites such as *Varroa* ingest infected host tissue that can contain viral replication intermediates, which would provide false positives during strand-specific RT-PCR assays (40, 51). More robust methods are, therefore, necessary to definitively identify which of the honey bee-infecting viruses can actively replicate in *V. destructor*. Electron microscopy to examine intracellular particle accumulation (38), fluorescent tagging of genetically engineered viral genomes (37), and *in situ* hybridization of negative-sense RNA replication intermediates (36) has been successfully used to assess DWV virus replication in mites; however, this becomes increasingly unfeasible when assessing replication of multiple viruses, distinct viral variants, or novel and uncharacterized virus species.

One efficient and unbiased method that can identify all replicating viruses within a sample is small RNA sequencing and examination of the antiviral RNA interference (RNAi) response. RNAi is one of the main antiviral defense mechanisms in arthropods (52). The antiviral RNAi pathway is triggered by the presence of dsRNA produced during viral replication, which is detected by the endonuclease Dicer-2, which then cleaves dsRNA into small interfering RNA (siRNA) 21–25 nt fragments (53). Cleaved siRNAs associate with the Argonaute protein AGO2, which delivers guide siRNAs to the RNA-induced silencing complex. The guide siRNAs bind to complementary sequences in their equivalent viral genomes, leading to the degradation of virus RNA and suppression of viral infection (54).

Insects typically produce sense and antisense viral siRNAs (vsiRNAs) of 20–23 nt (55–57), while other metazoans exhibit more divergent antiviral RNAi responses, with variation in vsiRNA length (23–30 nt) and polarity [e.g., negative strand bias; (58)]. Mites including *V. destructor* carry all genes required for the antiviral RNAi pathway (59); however, vsiRNA size profiles are distinct from those observed in insects. For example, both *A. mellifera* and *V. destructor* exhibit an active and abundant vsiRNA profile in response to infection with the negative-sense, ssRNA rhabdoviruses ARV-1 and -2, indicating active viral replication occurs within both species. However, *V. destructor* produces 24 nt vsiRNA fragments with a strong antisense bias, in contrast to the sense/antisense 22 nt vsiRNAs in *A. mellifera* (58). The contrasting vsiRNA size profiles between *A. mellifera* and *V. destructor* provide an opportunity to distinguish active antiviral responses in mites from those in bees and determine the viruses that actively replicate in *V. destructor* and are therefore genuine mite infections. Contrasting siRNA profiles have been previously used to distinguish between diverse hosts such as insects and fungi (55, 60). Applying this method broadly to *Varroa* populations carrying a range of honey bee-infecting viruses and strains, such as different genotypes of DWV, allows us to assess which of these are subject to biological vectoring.

In this study, we analyzed small RNA profiles of individual and pooled *V. destructor* mites from New Zealand, the Netherlands, South Africa, China, and the USA to identify active vsiRNA profiles indicative of replicating viruses. In particular, we were interested in determining whether multiple DWV genotypes undergo active replication in mites and whether other common honey bee-infecting viruses also produce active vsiRNA profiles in *V. destructor,* to better understand the status of mites as biological vectors of important honey bee pathogens.

## MATERIALS AND METHODS

### Sample collection and processing

*V. destructor* samples were collected from *A. mellifera* colonies in New Zealand (NZ), the Netherlands (NE), and South Africa (SA) during 2013–2018 (see Table S1 for sampling details). RNA was extracted from 15 individual mites and six pooled mite samples consisting of four mites to generate 21 new samples for small RNA sequencing (Table S1). Total RNA was extracted using TRIzol reagent (ThermoFisher). Whole individual mites were extracted in 100 µL of TRIzol and pooled mite sample volumes were scaled accordingly. Mites were homogenized in half the total TRIzol reagent with a sterile micropestle. The remaining TRIzol was added, followed by 1/5th volume of chloroform. RNA was precipitated from the aqueous phase with 1/2 volume of isopropanol and 1 µL glycogen and incubated at −20°C overnight. Following initial precipitation, RNA pellets were washed in 75% EtOH, and RNA resuspended in 6 µL H_2_O (single mites) or 25 µL H_2_O (pooled mites). RNA purity was confirmed with a Nanodrop spectrophotometer (ThermoFisher), and concentrations were quantified with the Qubit Bioanalyzer (ThermoFisher).

### Library preparation and sequencing

Small RNA (sRNA) libraries were generated using total RNA from single and pooled mites (100–600 ng) with the NEBNext Multiplex Small RNA Library Prep Kit (NEB) following the manufacturer’s protocol. Small RNA libraries were purified with the Monarch PCR Cleanup Kit (NEB) prior to size selection to obtain the correct fragment sizes for small RNA analysis. Libraries were mixed with Novex 5× Hi-Density TBE Sample Loading Dye (Thermo Fisher), then loaded into a 6% TBE acrylamide gel with the Quick-Load pBR322 DNA-MspI Digest ladder, and run for 65 minutes at 120 V. Gels were stained with SYBR Gold (Thermo Fisher) in 6% TBE buffer for 3 minutes, prior to size selection under a UV transilluminator. The region encompassing the small RNA size range of interest (15–35 nt, corresponding to the size ladder bands at 147–160 nt) was excised and purified by first passing through gel breaker tubes in DNA Gel elution buffer (NEB), incubated overnight at 4°C with shaking, then precipitated with 100% EtOH, 3 M sodium acetate (pH 5.5), and 2 µL glycogen for 4 hours at −80°C. The pellets were resuspended in 11 µL of TE buffer, and the sRNA libraries were shipped to the Australian Genome Research Facility for sequencing with Illumina HiSeq or Illumina NovaSeq 6000 (100 bp SE). Raw sequence reads generated from this study have been deposited at the NCBI Sequence Read Archive under the BioProject PRJNA986961 (Table S1).

### Small RNA composition analysis

In addition to the 21 new samples produced in this study, we obtained additional small RNA libraries of seven previously sequenced *V. destructor* samples from the NCBI Sequence Read Archive, two from our previous study of South African *V. destructor* (58), three from China (45), and two from the USA [(61) Table S1]. We initially examined all eight sRNA libraries from Kumar et al. (61); however, discarded data from six due to evidence of pseudoreplication (R. C. McKee, unpublished). Sequencing adaptors and low-quality reads were removed with TrimGalore (62), and read quality was evaluated with FastQC (63). To identify the sRNA composition of each sample, we first filtered our sequences for ribosomal RNA, because the proportion of rRNA reads captured during small RNA library synthesis is highly variable between samples (R. C. McKee, unpublished). We then performed sequential Bowtie2 (64) alignments, using the “--very sensitive local” parameters, changing -L to 15 to facilitate short read mapping. Reads were first aligned to the *V. destructor* genome (Vdes_3.0; Genbank accession GCA_002443255.1), then the *A. mellifera* genome (Amel_HAv3.1; GCA_003254395.2), and finally to a reference library containing virus genomes associated with *V. destructor*, adapted from Lester et al*.* [(16) Table S2]. For each consecutive alignment, unmapped reads were written to a separate fastq file, which was used for subsequent alignments. To identify shared reads between *V. destructor* and *A. mellifera*, libraries were first aligned to the *V. destructor* genome, and the aligned reads were written to a separate file, and then mapped to the *A. mellifera* genome, with any aligned reads corresponding to the number of reads shared between the two genomes. To ensure the number of shared reads was consistent, reciprocal alignments, starting with the *A. mellifera* genome, followed by the *V. destructor* genome, were performed. The resulting SAM files were converted to BAM files with Samtools (65). Geneious Prime 2022.2.2 (https://www.geneious.com/) was used to visualize the BAM file alignments.

### Consensus virus sequence assembly

To obtain strain-specific virus reference genomes for more accurate siRNA alignments, we attempted to generate consensus genome sequences of the viruses in individual samples to identify viral strain polymorphisms using two methods. First, we performed a *de novo* assembly of small RNA reads for each sample using Megahit [v 1.2.9 (66)]. Assembled contigs were examined for viral homology using a BLASTx search to a non-redundant viral protein database (NCBI; accessed August 2022). With this approach, we identified short fragments (~200–6,000 nt) of a number of known *V. destructor* and *A. mellifera* viruses, indicating that *de novo* assembly of our small RNA reads was generally insufficient for full viral genome reconstruction. However, in one small RNA library from the Netherlands (NE-6), BLASTx revealed two contigs with ~30% homology to Beihai horseshoe crab virus 1 (YP_009333375.1), indicating the presence of a putatively novel virus.

**Fig 1:**
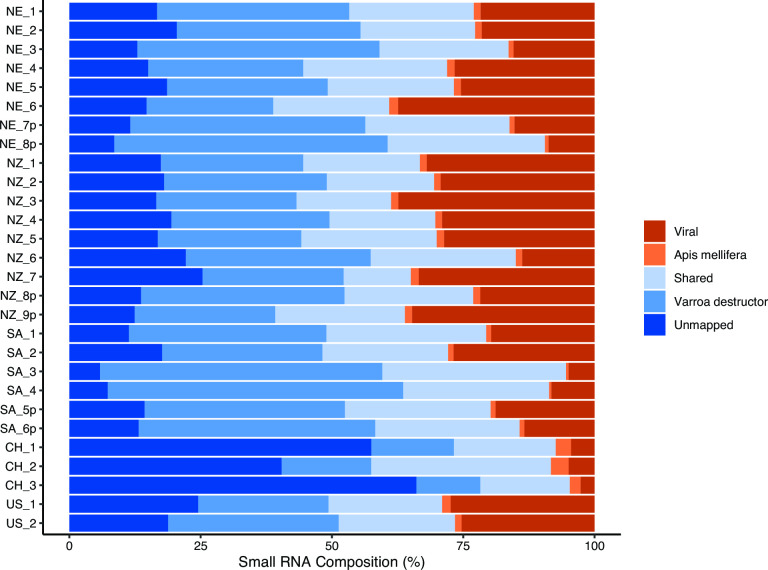
Small RNA composition of *V. destructor* samples. Each row indicates an individual mite sample; samples denoted “p” are derived from four pooled mites. Country is indicated (CH, China; US, United States). Reads were mapped to the *V. destructor* genome (Vdes_3.0; Genbank accession GCA_002443255.1) and then the *A. mellifera* genome (Amel_HAv3.1; GCA_003254395.2); shared reads indicate those able to align to both species’ genomes. Viral reads represent the total vsiRNA reads aligned to all viruses identified in each sample after the generation of sample-specific viral consensus genomes. The unmapped portion indicates reads that did not align to *V. destructor, A. mellifera*, or examined viral consensus genomes.

As we were not able to obtain sufficient strain information for all virus genomes from *de novo* assembly, we generated additional consensus sequences by performing a series of iterative Bowtie2 alignments of the short reads to reference viral genomes. We first performed an initial alignment to our reference virus genome library and generated consensus viral sequences for each sample in Geneious Prime using the “Generate Consensus Sequence” function. We set the consensus caller threshold in Geneious Prime to 0% “Majority,” to incorporate any sample-specific variants by allowing the fewest nucleotide ambiguities, and if there was no coverage, the reference sequence was called. Any within-sample nucleotide ambiguities were manually resolved by randomly selecting a representative alternate nucleotide according to the International Union of Pure and Applied Chemistry nucleotide naming nomenclature, prior to subsequent Bowtie2 alignments. For subsequent iterative alignments, the sRNA reads from each sample were aligned to their corresponding consensus viral reference sequences with Bowtie2, and new consensus viral sequences were synthesized using the aforementioned methods. Consensus generation and re-alignment were repeated for each sample until the increase in the number of reads aligning to the consensus sequences plateaued (two to nine iterations). After a final alignment of sRNA reads to the final consensus sequences for each sample, the total number of reads mapping to each virus was extracted from Geneious Prime. The “virus” component of our sRNA composition (Fig. 1) was calculated based on the total number of reads mapping to all viruses collectively after consensus strain iteration, divided by the total sRNA reads.

### Phylogenetic analysis of novel and known viruses

The novel virus sequence segment identified in one of our Netherlands mite samples (NE-6) was used to probe previously published RNA sequencing data from 27 *V*. *destructor* samples from New Zealand (16) and two from Tonga (58) to identify whether this novel virus was widespread and isolate a New Zealand variant. We assembled contigs *de novo* from the total RNA-seq reads in New Zealand and Tonga samples and used the contig representing our novel virus segment to identify the presence of homologous contigs using the “Map to reference function” (Geneious Prime). In addition, we used the RdRp-scan pipeline (67) and HMMER3 (68) to search for the missing genomic segment of our putative novel virus containing the RdRp coding region within the *de novo* assembled contigs. We also used the novel virus contig in Bowtie2 alignments to map unassembled reads. Furthermore, to investigate whether the novel virus segment was not exogenous, and instead potentially integrated into the *V. destructor* genome as an endogenous viral element, we examined published genomic sequencing reads of seven global Varroa *V. destructor* samples collected in 2020–2021 (NCBI, SRA: DRR292288, France; DRR303819, Iran; DRR303902 and DRR303896, Netherlands; DRR303719, Germany; SRR23020854, Japan; DRR303904, USA). We used the novel virus capsid segment for Bowtie2 alignments to determine whether gDNA reads mapped to the virus. We saw no evidence of reads mapping to the novel virus segment in these genomic libraries.

To perform phylogenetic analysis of the Netherlands and New Zealand strains of our novel virus segment, we obtained homologous virus genomes from current NCBI databases using BLASTx and performed protein alignments using MUSCLE (69). We used IQ-TREE to generate maximum likelihood phylogenies with 1,000 bootstraps and to determine the best-fit model (70). Treefiles were imported into FigTree (v1.4.4, http://tree.bio.ed.ac.uk/software/figtree/) and edited in Adobe Illustrator.

We also produced phylogenetic trees to examine nucleotide variation between global isolates of the viruses identified in our mite samples, using the strains assembled by Megahit or by consensus sequence generation after iterative alignments of the small RNA reads. After the final iteration of mapping, consensus sequences were generated for all viruses in each mite sample, leaving gaps over any regions that lacked coverage. Mite samples with lower virus levels returned consensus sequences that contained excessive gaps due to low coverage over some genomic regions and were thus excluded from further analysis. We constructed maximum likelihood phylogenetic trees using full-length genomes for the most highly prevalent viruses (ARV-1, ARV-2, VDV-2, VDV-3/-5, and DWV-A/-B), trimming regions with remaining short gaps in some samples. For DWV, we removed samples with evidence of recombinant variants, such as a DWV-B/A/B recombinant at the 5′ end of the genome in one of our Netherlands samples as previously characterized (20, 32). Nucleotide sequences were aligned using MUSCLE, and IQ_TREE was used to generate a maximum likelihood phylogeny as described above.

### Analysis of vsiRNA

All sRNA reads were imported into Rstudio using Rsamtools. Information about read strandedness (sense or antisense) and base composition (5′ first nucleotide) was extracted from .bam files using Rsamtools. Read length distributions and genomic coverage from alignments to initial and curated viral reference library for single-mite samples were plotted in Rstudio using ggplot2 and custom scripts.

## RESULTS

### Small RNA composition in mites

Mite small RNA libraries comprise 12%–56% of reads mapping to the *V. destructor* genome and 0.4%–3.4% of reads mapping to *A. mellifera* (Fig. 1). Due to the short nature of small RNA sequences and homology between genomes, 13%–35% of reads mapped to both genomes, though we assume the majority of these reads are derived from *V. destructor*. The number of reads mapping to the viral reference library varied between mite samples. Visual inspection of vsiRNA alignments to the viral reference genomes could clearly show when a virus was present within a sample; however, these alignments often had gaps, due to the highly polymorphic nature of RNA viruses, leading to siRNA reads with insufficient homology to viral reference genomes. To more accurately estimate the proportion of vsiRNA reads, consensus sequences for each virus were generated for each sample, and reads were re-aligned to the respective consensus genome sequences to capture the maximum number of viral reads. A significantly higher proportion of reads were found mapping to generated consensus genomes compared to the viral reference genomes (Fig. S1). Across all samples, the final estimate of aligned viral reads ranged from 2.6% to 37% (Fig. 1).

### Characterization of a novel *V. destructor* virus

**Fig 2:**
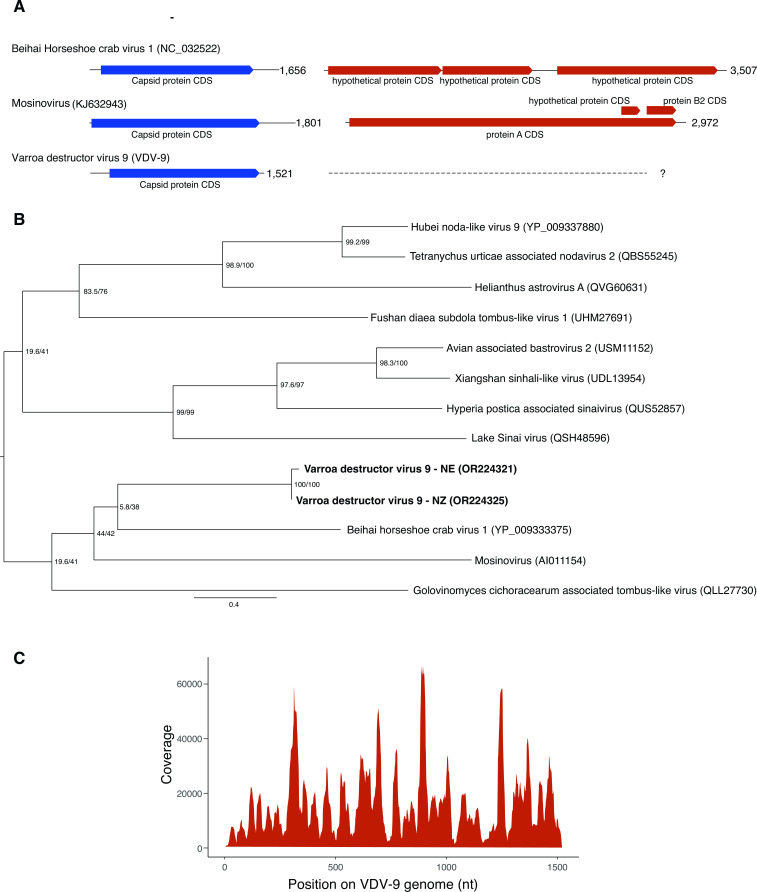
(**A**) Putative genome structure of Varroa destructor virus 9 compared to two distantly related bi-segmented viruses, Behai horseshoe crab virus 1, and mosinovirus. (**B**) Phylogenetic analysis of the capsid protein segment of Varroa destructor virus 9, identified in *V. destructor* samples from the Netherlands (NE; OR224321) and New Zealand (NZ; OR224325). Protein sequences of VDV-9 and homologous viruses obtained from NCBI were aligned using MUSCLE and trimmed for gaps, leaving a final length of 384 aa. The phylogenetic tree was generated using maximum likelihood in IQ-TREE (70), with the rtREV + F + I + G4 model, which had the optimal BIC score, as determined by ModelFinder (71). Branch supports were estimated using Ultrafast bootstrap approximation [UFBoot (72)] using 1,000 replicates. Support values shown are SH-aLRT and UFBoot supports. (**C**) Genome coverage of small RNA reads mapped to VDV-9 (sample NE-6).

We performed *de novo* assembly of the small RNA reads to generate longer contigs to assist with producing sample-specific viral consensus strains (see Materials and Methods). Assembled contigs were examined for homology to known viruses using BLASTx against a database containing all available protein viral sequences (NCBI) to identify the sequences with homology to viruses. In three samples from the Netherlands (NE-6, 7p, and -8p), we identified contigs that produced a full-length 6.2-kb genome with 75% identity to Bee macula-like virus (BMLV; NC_027631), indicating a novel variant (BMLV-NE 6–8; OR224322–OR224324; Fig. S2A). In addition, in the Netherlands sample (NE-6), we isolated two overlapping contigs that produced a 1.52-kb fragment with distant homology (31% amino acid identity) to the capsid protein of Beihai horseshoe crab virus 1 (YP_009333375.1; Fig. 2). Due to the lack of homology to any *V. destructor* or *A. mellifera* sequences, or to any previously discovered viruses in bees or mites, we assumed that this sequence represented a segment of a novel virus. To determine if this novel virus was present in other mites, we additionally examined published *V. destructor* RNA-seq data sets from New Zealand (16) and Tonga (58) and identified similar contigs with approximately 80% nucleotide identity to the novel virus in all transcriptomes examined (*n* = 27), suggesting this virus is common and widespread in *V. destructor*.

The distantly related Beihai horseshoe crab virus 1 genome contains two positive-sense ssRNA subgenomic segments: a 1.6-kb capsid segment with low homology to the fragment we identified in *V. destructor,* and a second 3.5-kb segment containing the RNA-dependent RNA polymerase [RdRp; Fig. 2A; (73)]. We were unable to isolate the expected second segment of the *V. destructor* novel virus among our assembled contigs using BLASTx, or via HMMER3 using the RdRp-scan pipeline (67). As RNA viruses are usually classified based on their RdRp homology (74), and we were only able to isolate the segment encoding the capsid protein in our novel virus, we were unable to assign a name that reflects taxonomy, so we named the novel virus “Varroa destructor virus 9” (Genbank accession numbers: OR224321 and OR224325). Phylogenetic analysis of the capsid protein sequence placed the Netherlands and New Zealand variants alongside the genomes of Beihai horseshoe crab virus 1 and mosinovirus, originally isolated from mosquitoes (75), with homology to nodaviruses. Both Beihai horseshoe crab virus 1 and mosinovirus possess a bipartite, positive-sense RNA genome, with mosinovirus containing an additional two sub-genomic segments (75). The capsid protein of this clade is distantly related to members of the *Tombus* and *Sinai* virus families, including the honey-bee infecting Lake Sinai virus (Fig. 2B).

### vsiRNA composition in mites

**Fig 3:**
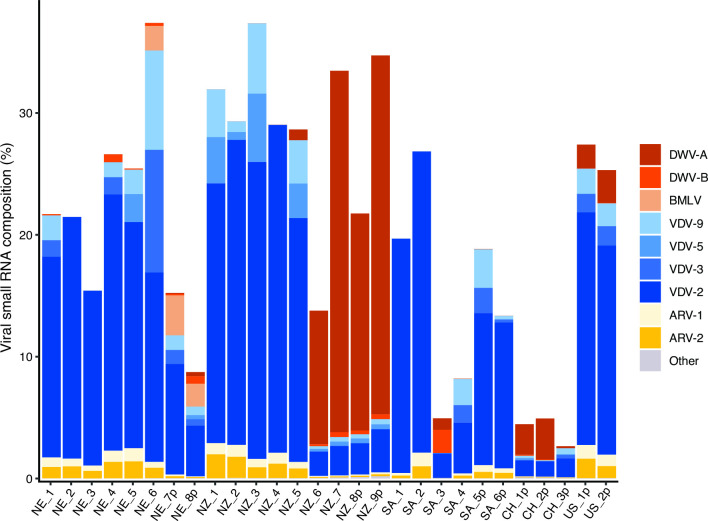
Composition of viruses within the viral small RNA portion of each sample. Each column represents an individual mite sample (named as in Fig. 1). *Y*-axis indicates the percentage of small RNA reads aligning to the virus, with each color indicating the proportion of total reads belonging to each virus species. “Other” indicates the low proportion of reads aligning to viruses not listed; such as SBV and BQCV in NZ-9p (VDV-3, Varroa destructor virus 3 and VDV-5, Varroa destructor virus 5).

We found evidence for a total of 10 viruses in mites across all of our samples, with the number of viruses present in individual mites ranging from three to nine viral species (Fig. 3; Table 1). Positive-sense ssRNA viruses accounted for 8 of the 10 viruses, along with two negative-sense ssRNA viruses (ARV-1 and ARV-2). VDV-2 was the most prevalent virus, present in all *V. destructor* samples (*n* = 28), followed by ARV-1 and ARV-2, which were present in all but one sample from South Africa (SA-3). VDV-2 was also the most abundant virus, accounting for between 7% and 98% of vsiRNA reads, followed by DWV-A (0%–89%) and DWV-B (0%–39%). DWV abundance varied considerably between samples and was particularly high in four DWV-A containing mites from NZ (NZ-6, 7, 8p, and 9p), accounting for 80%–89% of viral reads.

**TABLE 1:** Summary of the replicating viruses identified in *Varroa destructor^a^*

Virus name/abbreviation		Genome polarity	Positive samples	Virus replication
Deformed wing virus A	DWV-A	+ssRNA	19	+/–
Deformed wing virus B	DWV-B	+ssRNA	7	+/–
Sacbrood virus	SBV	+ssRNA	1	–
Black queen cell virus	BQCV	+ssRNA	1	–
Bee macula-like virus	BMLV	+ssRNA	4	+/–
Apis rhabdovirus 1	ARV-1	−ssRNA	27	+
Apis rhabdovirus 2	ARV-2	−ssRNA	27	+
Varroa destructor virus 2	VDV-2	+ssRNA	28	+
Varroa destructor virus 3	VDV-3	+ssRNA	13	+
Varroa destructor virus 5	VDV-5	+ssRNA	13	+
Varroa destructor virus 9	VDV-9	+ssRNA	23	+

^*a*^ “+” indicates viruses with sRNA profiles consistent with active degradation and virus replication; “−” indicates viruses with sRNA read profiles that are consistent with random degradation; “+/−” indicates viruses that exhibit patterns consistent with both active and random degradation.

### Phylogenetic clustering of consensus viral sequences

We performed phylogenetic analysis of the most prevalent viruses across individual samples (DWV-A/-B, Fig. S2B; ARV-1, ARV-2, VDV-2, and VDV-3/–5, Fig. S3) by aligning the consensus sequences generated after iterative sRNA read mapping. Most viruses clustered broadly according to the location with some individual exceptions (Fig. S2B and S3). As VDV-3 and VDV-5 share approximately 75% nucleotide identity, they were analyzed in the same tree. The distributions of VDV-3 and -5 varied with NZ samples containing a VDV-5 variant (~92% identity to VDV-5 reference), US and SA samples containing VDV-3, and the NE and CH samples showing evidence of both VDV-3 and VDV-5 variants (Fig. S3C).

Three separate VDV-2 consensus strains were generated for each sample, based on the three currently available reference genomes in NCBI (Israel, KX578271; UK, MK795517; and China, MW590582), which show between 75% and 83% nucleotide identity to each other. High coverage of all three reference strains in most samples (File S1) indicates the presence of multiple VDV-2 strains within the same mite. Consensus sequence alignments clustered according to the reference VDV-2 sequence from which the consensus was originally based, and then by location (Fig. S3D).

### vsiRNA profiles reveal active degradation of *Varroa-*infecting viruses

**Fig 4:**
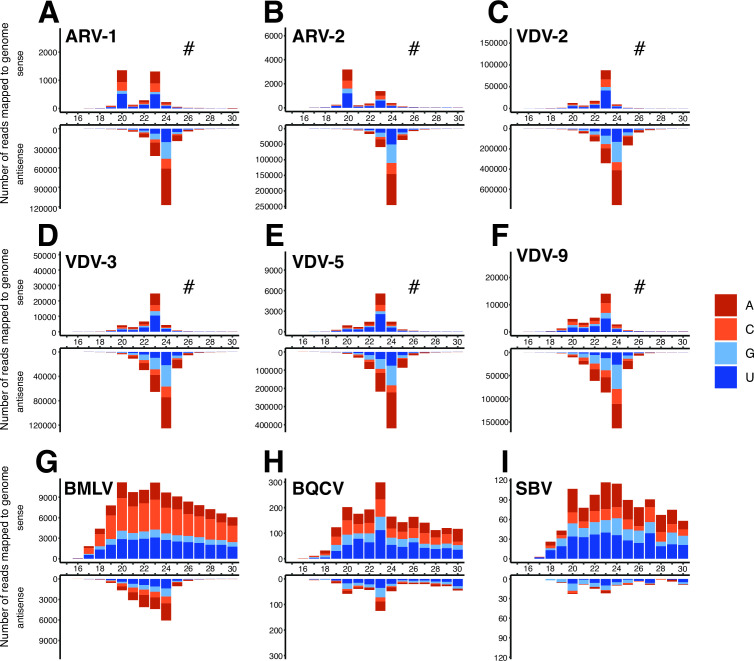
Size profiles of viral small RNA reads mapping to (**A**) Apis rhabdovirus 1; (**B**) Apis rhabdovirus 2; (**C**) Varroa destructor virus 2; (**D**) Varroa destructor virus 3 (VDV-3); (**E**) Varroa destructor virus 5 (VDV-5); (**F**) Varroa destructor virus 9; (**G**) bee macula-like virus; (**H**) black queen cell virus; and (**I**) sacbrood virus. Representative profiles are shown from samples chosen at random (NZ-4: A, C; NZ-1, B; US-1p: D, F; NZ-3: E; NE-8p: G; NZ-9p: H, I) with near identical patterns observed for all mites infected with the above viruses (see Fig. S4 for additional examples). # indicates the sense *Y*-axis has been scaled to aid in visualizing any size peaks.

**Fig 5:**
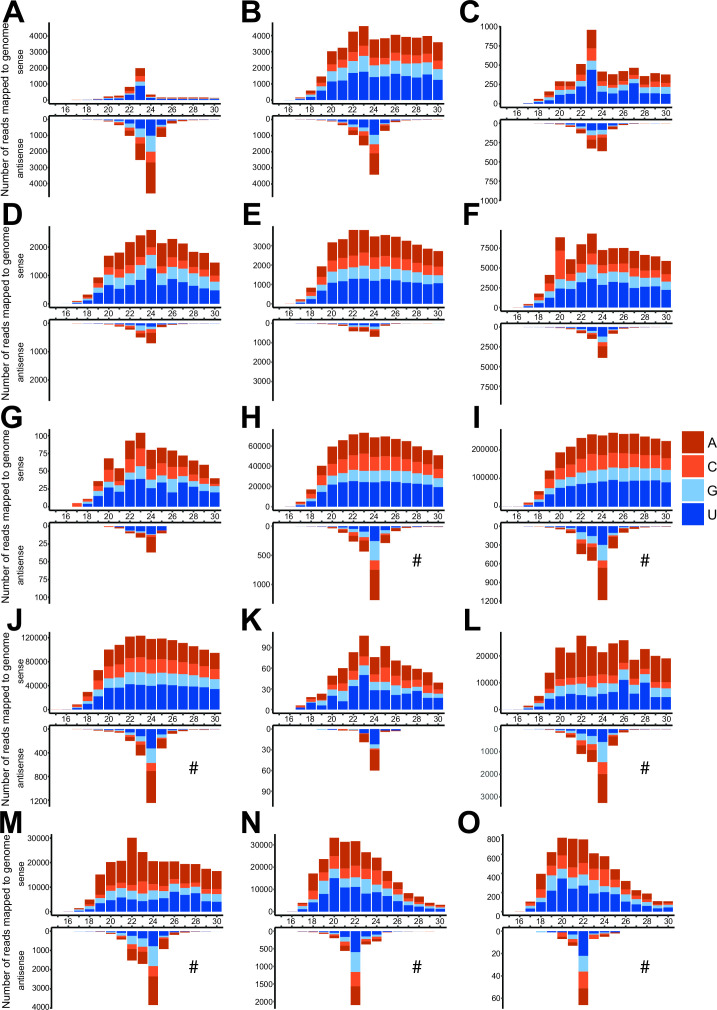
Size profiles of viral small RNA reads mapping to deformed wing virus B (**A–E**) and deformed wing virus A (**F–O**) from (A) NE-1; (**B**) NE-4; (**C**) NE-5; (**D**) NE-6; (**E**) NE-8p; (**F**) NZ-5; (**G**) NZ-2; (**H**) NZ-6; (**I**) NZ-7; (**J**) NZ-8p; (**K**) SA-5p; (**L**) US-1p; (**M**) US-2p, (**N**) CH-2p; and (**O**) CH-3p. DWV antisense genome coverage plots are presented in Fig. S5 for three representative DWV-B samples (NE-1, NE-4, and NE-8p) and three representative DWV-A samples (NZ-5, US-2p, and CH-2). The antisense profiles of DWV-B in samples C and D above (NE-5 and -6) did not have adequate antisense coverage for genome plots. # indicates the antisense *Y*-axis has been scaled to aid in visualizing any size peaks.

We examined the size profiles of viral small RNA reads mapping to all viruses present in our samples (Fig. 4; Fig. S4), including DWV-A and -B (Fig. 5). The vsiRNA reads mapping to known *V. destructor*-infecting viruses (ARV-1 and -2, VDV-2, VDV-3, and VDV-5) have a clear antisense bias with 60%–99% of reads in the antisense orientation, with some variation between viruses (Fig. 4A through E; Fig. S4; File S1). Most of the observed antisense reads have a size distribution peak at 24 nt, which is consistent between the −ssRNA viruses ARV-1 and -2 (Fig. 4A and B) and the +ssRNA viruses VDV-2,-3, and -5 (Fig. 4C through E). In addition to the 24 nt antisense bias, most viruses also have identifiable sense peaks at 20 and 23 nt, with the 20 nt peak more prominent in −ssRNA viruses and the 23nt peak more prominent in +ssRNA viruses. The novel virus, VDV-9, has an identical pattern to other *V. destructor*-infecting +ssRNA viruses with a strong 24 nt antisense peak, and a detectable 23 nt sense peak, indicating it is an actively replicating virus within *V. destructor*, which can be degraded by the antiviral RNAi pathway (Fig. 4F).

Honey bee-associated viruses (BMLV, BQCV, and SBV) have more ambiguous degradation profiles. BMLV has a mixed profile with a clear antisense 24 nt peak, though the high proportion of sense reads appears to be a result of non-specific degradation (27%–84%), which corresponds to the genome polarity of BMLV (+ssRNA; Fig. 4G; Fig. S4G; File S1). BQCV and SBV were identified in one pooled mite sample (NZ-9p), but these viruses are in low abundance, lack a 24 nt antisense peak, and have a high proportion of sense reads (>80%), consistent with non-specific degradation (Fig. 4H and I).

While most *V. destructor*-infecting viruses display consistent RNAi-mediated degradation profiles between all mite samples (Fig. 4A through F; Fig. S4), DWV shows variable profiles between individual mites (Fig. 5). In one DWV-B-infected sample from the Netherlands (NE-1), there is a large proportion of antisense vsiRNA reads (69%) with a clear 24 nt peak and an additional 23 nt sense peak (Fig. 5A), similar to the RNAi-mediated degradation profiles of other +ssRNA viruses (Fig. 4C through F). Other DWV-infected mites have evidence of a 24 nt antisense peak along with a 23 nt sense peak (e.g., Fig. 5C through F), though with a higher proportion of sense reads. In particular, in samples where DWV is highly abundant (Fig. 3), the majority of reads aligning to DWV have a positive sense distribution consistent with DWV genome polarity (+ssRNA), and a much lower proportion of antisense reads, though with an observable antisense peak at 24 nt in most samples (Fig. 5H through J and L through N). DWV genome coverage plots for antisense reads mapping to DWV-B (Fig. S5A through C) and DWV-A (Fig. S5D through F) indicate that in samples with sufficient coverage, antisense reads span the full length of the genome. Interestingly, two mite samples from China (CH-2p and CH-3p, Fig. 5N and O) show a 22 nt antisense peak, consistent with the mites having ingested honey bee-produced vsiRNAs (56, 58). Collectively, the degradation profiles of DWV-A and -B genotypes indicate a mixed mode of RNAi-mediated and non-specific degradation, suggesting that both genotypes have evidence of active replication in mites, but that a considerable viral load is also non-specifically degraded, either due to ingested honey bee tissues or due to unchecked viral replication and escape from RNAi control within mite cells (Fig. 5).

### Replicating viruses in *V. destructor*

Based on the active vsiRNA profiles identified, we summarize the replication status of all viruses present in our *V. destructor* samples (Table 1). A total of eight viruses (including both DWV-A/-B strains) have RNAi-mediated degradation profiles indicative of viral replication in *V. destructor*.

## DISCUSSION

*V. destructor* is associated with an array of viruses, many of which are due to its role as a parasite of *A. mellifera* and a viral vector of bee-infecting viruses. Due to the critical role of *V. destructor* in the transmission of bee viruses, it is important to understand the mechanisms by which mites achieve antiviral immunity and to identify viruses that are replicating within mites. We took advantage of the differences in antiviral siRNA profiles between *A. mellifera* and *V. destructor* to comprehensively characterize vsiRNA profiles in individual mites to identify replicating viruses and determine whether *V. destructor* is a genuine host of these viruses. We identified eight actively replicating viruses in *V. destructor,* based on host-specific vsiRNA profiles obtained by examining small RNA from mites sampled globally. Most notably, we provide direct evidence for the replication of DWV-A and -B within mites, suggesting that *Varroa* is a biological vector of both genotypes. Our analysis of vsiRNA profiles in *V. destructor* identifies clear antiviral degradation with viruses from both positive-sense ssRNA and negative-sense ssRNA families showing a 24 nt antisense peak and additional minor sense peaks. The mixed RNAi-mediated/non-specific degradation pattern observed toward DWV in some mite samples was also evident toward another honey-bee-associated virus, BMLV, but not toward SBV and BQCV. We also present evidence of a novel, bi-segmented +ssRNA virus that is widespread in *V. destructor*, VDV-9, which produces a 24 nt vsiRNA degradation profile and is, therefore, likely to be an actively replicating virus in mites.

For most viruses present in our mite samples, we observed vsiRNA profiles with a 24 nt antisense bias (Fig. 4). Polarity biases during RNAi-mediated antiviral defense are not unique to *V. destructor*, as similar polarity biases have been observed in *C. elegans* (76), *Aedes aegypti* (77), and a range of other metazoans (78, 79). We categorized viruses with a distinct 24 nt antisense peak as replicating viruses within *V. destructor* and identified eight viruses replicating within our samples based on this criterion (Table 1). Notably, all *V. destructor* samples contained VDV-2, and all but one sample had ARV-1 and ARV-2 (Fig. 3; Table 1), which are three viruses known to be highly prevalent in *V. destructor* (16, 39, 42, 43). We saw evidence of individual mites containing multiple VDV-2 strains (Fig. S3D), as previously observed in New Zealand *V. destructor* samples (16), though this is difficult to resolve conclusively using small RNA data, due to short read lengths and nucleotide similarities between strains. Based on the high prevalence of ARV-1 and ARV-2 in mites, and low prevalence in honey bees and other bee species (20, 80), there is accumulating evidence that *V. destructor* is the primary host for these −ssRNA rhabdoviruses instead of *A. mellifera*, and that these viruses frequently co-occur in both bees (81) and mites. Additional rhabdoviruses have also been identified in *Varroa jacobsoni* (82), as well as within *A. mellifera* and *Apis cerana* in China (45), indicating substantial diversity in the −ssRNA rhabdovirus clade across bees and their mite parasites.

The novel viral genome identified in this study, Varroa destructor virus 9, was also present in 23 (82%) of our mite samples (Fig. 3; Table 1), with vsiRNA profiles consistent with viral replication (Fig. 4F). The genomic segment identified in our samples has low homology to the capsid protein of other +ssRNA bi-segmented viruses including Behai horseshoe crab virus 1 and mosinoviruses (Fig. 2), yet we were unable to isolate the RdRp-coding segment in our data. It is, therefore, likely that a portion of the “unmapped” small RNA reads (Fig. 1) belong to our as yet unidentified RdRp-containing fragment or indeed to other as yet unidentified viruses in these samples. It is possible that the predicted VDV-9 segment containing the RdRp-coding region lacks sufficient homology to currently known viruses and, therefore, cannot be identified by available methods. Identifying highly divergent viral sequences from the “dusk matter” of metatranscriptomic data sets is a common challenge, and virus-derived small RNA has been successful in identifying novel lineages that lack sufficient homology to known viruses (55, 83). However, our RdRp-specific HMM search, which can identify homology as low as 10% (67), did not yield any novel contigs with RdRp homology from our small RNA samples or additional transcriptomes from New Zealand *V. destructor*. Alternatively, the capsid segment we identified here might represent the outcomes of a novel viral reassortment (84), where this segment has become associated with another, already-known virus present in mites. The vast majority of viruses identified in *Varroa* have been characterized at sequence level only, with no knowledge of viral particle structure or understanding of disease phenotypes, thus it is not unfeasible that viral segments may be missing from previously characterized viruses. There is clearly more to be done to understand the role of VDV-9 as a member of the *V. destructor* virome. More broadly, we require a deeper understanding of how the diverse and complex virome present in individual mites, which contain between three and nine viruses, impacts *V. destructor* biology.

Although BQCV and SBV are commonly among the most prevalent viruses identified in honey bee samples globally (15, 45, 85–87), they were detected in only one of our *V. destructor* samples (NZ-9p). Negative-strand RT-PCR assays for BQCV have previously implied active replication occurs in *V. destructor*, suggesting it could be a biological vector for this virus (88); however, it is possible that BQCV negative-strand RT-PCR may have detected honey bee infected tissue within *V. destructor*. From our limited availability of positive samples (*n* = 1), the distribution of small RNA reads mapping to BQCV is not consistent with replication and shows a random degradation profile (Fig. 4H). Similarly, SBV vsiRNA read profiles were also inconsistent with replication (Fig. 4I), suggesting that *V. destructor* is not a biological vector of BQCV or SBV, though it may still transmit viral particles passively as a mechanical vector. Analysis of small RNA profiles of additional mites containing BQCV and SBV is necessary before this can be conclusively stated. The lack of *V. destructor* samples with BQCV and SBV supports the hypothesis that viruses such as these, which show high lethality in juvenile life stages (89, 90) are incompatible with the *V. destructor* life cycle, leading to selection against vectoring by mites (91, 92).

Because DWV is the main virus associated with colony declines, the question of whether *V. destructor* is a biological vector and a true host for DWV has been a topic of great interest in honey bee research (35, 50, 88, 93). To add to growing experimental data that indicate active replication of DWV-B and recombinant DWV genotypes within *Varroa* tissues (36, 37), we provide direct evidence of DWV-A and -B replication in *V. destructor* via the presence of RNAi-mediated antiviral degradation profiles.

Multiple DWV-infected mites show vsiRNA profiles with distinct 24 nt antisense peaks (Fig. 5), as seen in actively replicating +ssRNA viruses in *V. destructor* (Fig. 4C through F), indicating that there is active, RNAi-mediated degradation and, therefore, viral replication of DWV within mites. This active degradation profile was present in mite samples containing DWV-A and -B (Fig. 5A through M). Some samples show a mixed profile with the 24 nt antisense peak and a higher proportion of sense reads showing non-specific degradation, which could indicate that individual mites have low levels of DWV replication, and the vsiRNA signal is overwhelmed by the high proportion of ingested DWV from infected *A. mellifera* tissue, resulting in a random degradation profile that swamps the proportion of antisense reads. Mites with lower DWV loads tended to have clearer active degradation profiles, in support of this hypothesis. These differing degradation patterns could reflect mites at various stages of infection. It is likely that the use of whole crushed mites yielded higher positive-sense, non-specific degraded reads, because small RNA from all tissues was represented, including from the digestive tract, which contains degradation products of ingested viruses. Of note, two samples (Fig. 5N and O) show clear 22 nt antisense peaks, evidence that these mites have ingested honey bee-produced vsiRNAs rather than generated their own antiviral response. This outcome shows the sensitivity of our method in detecting *V. destructor*-generated antiviral small RNA fragments, which have a clear size difference (24 nt) to *A. mellifera-*generated fragments (22 nt). The ability of mites to vector DWV-A decreases following experimental feeding on pupae containing lower DWV-A loads (40), suggesting that the majority of vectored viral particles are acquired by feeding on infected hosts (mechanical) rather than replication within mites (biological), which is consistent with the mixed-mode degradation profiles that we observe in the majority of our samples (Fig. 5).

Alternatively, the high positive sense reads in some samples could indicate that actively replicating DWV virus has overwhelmed the RNAi response and exceeded the mite’s immune capacity to achieve sufficient viral control. Mites with high DWV-B loads show reduced lifespan compared to mites with high DWV-A, which suggests that *Varroa* viability is impacted by DWV load and that DWV-A infections may be more efficiently suppressed by mite antiviral defenses, leading to improved *Varroa* lifespan (94).

*In situ* hybridization shows that replicating DWV-B is localized to gut epithelial tissue and salivary glands, reinforcing the notion that DWV replicates in key tissues of *V. destructor*, which are compatible with biological vectoring (36). In the same study, DWV-A replication was not detected by *in situ* hybridization, though the authors did not confirm the presence of DWV-A in their source population, thus DWV-A is likely to be absent from these samples. Our data suggest that there is no strain specificity regarding DWV replication. While individual mite DWV vsiRNA profiles vary considerably along with variation in DWV loads, overall, the presence of some degree of siRNA-mediated antiviral response to DWV-A (Fig. 5E through M) and DWV-B (Fig. 5A through D) in multiple individual mites indicates that *V. destructor* is a genuine host for both DWV-A and -B genotypes.

Currently, the mechanism underpinning the 24 nt antisense peak that differentiates the *V. destructor* vsiRNA profile from the typical 21–22 nt sense/antisense profile observed in honey bees and other insects is unknown. In the roundworm, *Caenorhabditis elegans*, an abundance of antisense siRNA reads are produced during secondary amplification of exogenous siRNA by an RNA-directed-RNA-polymerase (74). These secondary siRNAs are distinct from primary siRNAs produced by the Dicer pathway and enhance antiviral immunity. Notably, however, the size profiles of secondary siRNAs in *C. elegans* are 22 nt and they possess a 5′ triphosphate, which makes them refractory to standard library preparations (76, 95). It will be interesting to determine whether 24 nt siRNA biogenesis in Varroa has any similarities to that in nematodes, or if they are made through a different biogenesis pathway. RdRp orthologs are largely absent from most insects including honey bees (96); however, they have been identified in members of the Acari subclass (ticks and mites), with four identified in the *V. destructor* genome (59). The role of RdRps during RNAi-mediated antiviral defense has not been studied in *Varroa*, but some work has been done within other members of Acari. Like *V. destructor,* the black-legged tick *Ixodes scapularis* has four RdRps within its genome. However, in *I. scapularis*, RNAi-mediated antiviral immunity generates vsiRNAs, which are 22 nt in length, with no clear polarity bias (97) suggesting that RdRps are unlikely to be involved in secondary siRNA synthesis in this species. The *V. destructor* genome contains multiple Dicer-2 and Ago-2 isoforms (59), and gene expansions in these other components involved in siRNA-mediated antiviral immunity may have functionally distinct roles in mites. It is possible that the presence of multiple *Dicer-2* homologs in the *V. destructor* genome results in different small RNA populations in a similar mechanism to plant antiviral defense, where 21, 24, and 22 nt siRNA have been observed to be generated by different *Dicer* genes (98, 99). Currently, it is unclear which of the RNAi pathway components produces *V. destructor*’s uniquely sized antisense siRNA profile in response to viral replication, or if mite RdRps are used to enhance antiviral immunity in a similar mechanism to *C. elegans*. Nevertheless, the alternative antiviral RNAi mechanism present in mites has allowed us to successfully identify replicating viruses within *V. destructor*, most notably with DWV.

Our study shows that *V. destructor* harbors a core virome that is abundant and diverse, which is actively targeted by the mite’s antiviral RNAi pathway. Our study has added to this diversity with the identification of a novel, capsid-encoding segment of a putatively bi-segmented +ssRNA virus, VDV-9, with homology to tombusviruses and Sinai-like viruses (Fig. 2). It is presently unclear whether mite-infecting viruses cause pathology in *V. destructor*. Enhanced antiviral protection may be required to protect mites against their own viruses, or to withstand the pathogenic impact of the viruses that mites vector to honey bees. Our results suggest that the role of *V. destructor* in virus transmission is mixed and involves both active and passive transmission. Ultimately, our results indicate that honey bee viruses like DWV-A, DWV-B, and BMLV have adapted to replicate within mites, as a result of viral spillover from the honey bee host. Host-parasite viral spillover is, therefore, a key contributing factor to the increasing impact of viruses on honey bee health as a result of the global spread of *V. destructor*.

## Data Availability

Raw sequence reads generated from this study have been deposited at the NCBI Sequence Read Archive under the BioProject PRJNA986961 (Table S1).

## SUPPLEMENTAL MATERIAL

Supplemental tables and figures jvi.01149-23-s0001.pdf Tables S1, S2 and Figures S1, S2, S3, S4, S5.

Supplemental file 1 jvi.01149-23-s0002.xlsx Spreadsheet showing the numbers and proportions of sense and antisense reads mapping to the viruses identified in this study.

ASM does not own the copyrights to Supplemental Material that may be linked to, or accessed through, an article. The authors have granted ASM a non-exclusive, world-wide license to publish the Supplemental Material files. Please contact the corresponding author directly for reuse.

## References

[1] Koch KG, Jones T-K, Badillo-Vargas IE. 2020. Chapter 26 - Arthropod vectors of plant viruses, p 349–379. In Awasthi LP (ed), Applied plant Virology. Academic Press.

[2] Mellor PS. 2000. Replication of arboviruses in insect vectors. J Comp Pathol 123:231–247. doi:10.1053/jcpa.2000.0434 11041993

[3] Oliveira JH, Bahia AC, Vale PF. 2020. How are arbovirus vectors able to tolerate infection. Dev Comp Immunol 103:103514. doi:10.1016/j.dci.2019.103514 31585195

[4] Calderone NW. 2012. Insect pollinated crops, insect pollinators and US agriculture: trend analysis of aggregate data for the period 1992-2009. PLoS One 7:e37235. doi:10.1371/journal.pone.0037235 22629374 PMC3358326

[5] Neumann P, Carreck NL. 2010. Honey bee colony losses. J of Api Res 49:1–6. doi:10.3896/IBRA.1.49.1.01

[6] Oldroyd BP. 1999. Coevolution while you wait: Varroa jacobsoni, a new parasite of Western honeybees. Trends Ecol Evol 14:312–315. doi:10.1016/s0169-5347(99)01613-4 10407428

[7] Anderson DL, Trueman JWH. 2000. Varroa jacobsoni (Acari: varroidae) is more than one species. Exp Appl Acarol 24:165–189. doi:10.1023/a:1006456720416 11108385

[8] Roberts JMK, Anderson DL, Tay WT. 2015. Multiple host shifts by the emerging honeybee parasite, Varroa jacobsoni*.* Mol Ecol 24:2379–2391. doi:10.1111/mec.13185 25846956

[9] Rosenkranz P, Aumeier P, Ziegelmann B. 2010. Biology and control of Varroa destructor. J Invertebr Pathol 103 Suppl 1:S96–119. doi:10.1016/j.jip.2009.07.016 19909970

[10] Ramsey SD, Ochoa R, Bauchan G, Gulbronson C, Mowery JD, Cohen A, Lim D, Joklik J, Cicero JM, Ellis JD, Hawthorne D, vanEngelsdorp D. 2019. Varroa destructor feeds primarily on honey bee fat body tissue and not hemolymph. Proc Natl Acad Sci U S A 116:1792–1801. doi:10.1073/pnas.1818371116 30647116 PMC6358713

[11] Bowen-Walker PL, Gunn A. 2001. The effect of the ectoparasitic mite, Varroa destructor on adult worker honeybee (Apis mellifera) emergence weights, water, protein, carbohydrate, and lipid levels. Entomologia Experimentalis et App 101:207–217. doi:10.1046/j.1570-7458.2001.00905.x

[12] Traynor KS, Mondet F, de Miranda JR, Techer M, Kowallik V, Oddie MAY, Chantawannakul P, McAfee A. 2020. Varroa destructor: a complex parasite, crippling honey bees worldwide. Trends Parasitol 36:592–606. doi:10.1016/j.pt.2020.04.004 32456963

[13] Ribière M, Ball B, Aubert MFA. 2008. Natural history and geographical distribution of honey bee viruses. In Aubert M, Ball B, Fries I, Moritz R, Milani N, Bernardinelli I (ed), Virology and the honey bee. EEC Publications, Luxembourg.

[14] Bowen-Walker PL, Martin SJ, Gunn A. 1999. The transmission of deformed wing virus between honeybees (Apis mellifera L.) by the ectoparasitic mite Varroa jacobsoni oud. J Invertebr Pathol 73:101–106. doi:10.1006/jipa.1998.4807 9878295

[15] Mondet F, de Miranda JR, Kretzschmar A, Le Conte Y, Mercer AR, Schneider DS. 2014. On the front line: quantitative virus dynamics in honeybee (Apis Mellifera L.) colonies along a new expansion front of the parasite Varroa destructor. PLoS Pathog 10:e1004323. doi:10.1371/journal.ppat.1004323 25144447 PMC4140857

[16] Lester PJ, Felden A, Baty JW, Bulgarella M, Haywood J, Mortensen AN, Remnant EJ, Smeele ZE. 2022. Viral communities in the parasite Varroa destructor and in colonies of their honey bee host (Apis mellifera) in New Zealand. Sci Rep 12:8809. doi:10.1038/s41598-022-12888-w 35614309 PMC9133037

[17] Martin SJ, Highfield AC, Brettell L, Villalobos EM, Budge GE, Powell M, Nikaido S, Schroeder DC. 2012. Global honey bee viral landscape altered by a parasitic mite. Science 336:1304–1306. doi:10.1126/science.1220941 22679096

[18] Di Prisco G, Annoscia D, Margiotta M, Ferrara R, Varricchio P, Zanni V, Caprio E, Nazzi F, Pennacchio F. 2016. A Mutualistic symbiosis between a parasitic mite and a pathogenic virus undermines honey bee immunity and health. Proc Natl Acad Sci U S A 113:3203–3208. doi:10.1073/pnas.1523515113 26951652 PMC4812730

[19] Wilfert L, Long G, Leggett HC, Schmid-Hempel P, Butlin R, Martin SJM, Boots M. 2016. Deformed wing virus is a recent global epidemic in honeybees driven by Varroa mites. Science 351:594–597. doi:10.1126/science.aac9976 26912700

[20] Norton AM, Remnant EJ, Tom J, Buchmann G, Blacquiere T, Beekman M. 2021. Adaptation to vector‐based transmission in a honeybee virus. J Anim Ecol 90:2254–2267. doi:10.1111/1365-2656.13493 33844844

[21] Barroso-Arévalo S, Fernández-Carrión E, Goyache J, Molero F, Puerta F, Sánchez-Vizcaíno JM. 2019. High load of deformed wing virus and Varroa destructor infestation are related to weakness of honey bee colonies in Southern Spain. Front Microbiol 10:1331. doi:10.3389/fmicb.2019.01331 31258521 PMC6587608

[22] Highfield AC, El Nagar A, Mackinder LCM, Noël L-L, Hall MJ, Martin SJ, Schroeder DC. 2009. Deformed wing virus implicated in overwintering honeybee colony losses. Appl Environ Microbiol 75:7212–7220. doi:10.1128/AEM.02227-09 19783750 PMC2786540

[23] Martin SJ, Brettell LE. 2019. Deformed wing virus in honeybees and other insects. Annu Rev Virol 6:49–69. doi:10.1146/annurev-virology-092818-015700 31185188

[24] Mordecai GJ, Wilfert L, Martin SJ, Jones IM, Schroeder DC. 2016. Diversity in a honey bee pathogen: first report of a third master variant of the deformed wing virus quasispecies. ISME J 10:1264–1273. doi:10.1038/ismej.2015.178 26574686 PMC5029213

[25] de Miranda JR, Brettell LE, Chejanovsky N, Childers AK, Dalmon A, Deboutte W, de Graaf DC, Doublet V, Gebremedhn H, Genersch E, et al.. 2022. Cold case: the disappearance of Egypt bee virus, a fourth distinct master strain of deformed wing virus linked to honeybee mortality in 1970’s Egypt. Virol J 19:12. doi:10.1186/s12985-022-01740-2 35033134 PMC8760790

[26] Hasegawa N, Techer MA, Adjlane N, Al-Hissnawi MS, Antúnez K, Beaurepaire A, Christmon K, Delatte H, Dukku UH, Eliash N, El-Niweiri MAA, Esnault O, Evans JD, Haddad NJ, Locke B, Muñoz I, Noël G, Panziera D, Roberts JMK, De la Rúa P, Shebl MA, Stanimirovic Z, Rasmussen DA, Mikheyev AS. 2023. Evolutionarily diverse origins of deformed wing viruses in Western honey bees. Proc Natl Acad Sci U S A 120:e2301258120. doi:10.1073/pnas.2301258120 37339224 PMC10293827

[27] Paxton RJ, Schäfer MO, Nazzi F, Zanni V, Annoscia D, Marroni F, Bigot D, Laws-Quinn ER, Panziera D, Jenkins C, Shafiey H. 2022. Epidemiology of a major honey bee pathogen, deformed wing virus: potential worldwide replacement of genotype A by genotype B. Int J Parasitol Parasites Wildl 18:157–171. doi:10.1016/j.ijppaw.2022.04.013 35592272 PMC9112108

[28] Ryabov Eugene V., Childers AK, Chen Y, Madella S, Nessa A, vanEngelsdorp D, Evans JD. 2017. Recent spread of Varroa destructor virus-1, a honey bee pathogen, in the United States. Sci Rep 7:17447. doi:10.1038/s41598-017-17802-3 29234127 PMC5727227

[29] Kevill JL, Stainton KC, Schroeder DC, Martin SJ. 2021. Deformed wing virus variant shift from 2010 to 2016 in managed and Feral UK honey bee colonies. Arch Virol 166:2693–2702. doi:10.1007/s00705-021-05162-3 34275024 PMC8421296

[30] Ryabov E.V, Wood GR, Fannon JM, Moore JD, Bull JC, Chandler D, Mead A, Burroughs N, Evans DJ. 2014. A virulent strain of deformed wing virus (DWV) of honeybees (Apis mellifera) prevails after Varroa destructor-mediated, or in vitro, transmission. PLoS Pathog 10:e1004230. doi:10.1371/journal.ppat.1004230 24968198 PMC4072795

[31] Cornman RS. 2017. Relative abundance of deformed wing virus, Varroa destructor virus 1, and their recombinants in honey bees (Apis mellifera) assessed by kmer analysis of public RNA-Seq data. J Invertebr Pathol 149:44–50. doi:10.1016/j.jip.2017.07.005 28743669

[32] Norton AM, Remnant EJ, Buchmann G, Beekman M. 2020. Accumulation and competition amongst deformed wing virus genotypes in naive Australian honeybees provides insight into the increasing global prevalence of genotype B. Front Microbiol 11:620. doi:10.3389/fmicb.2020.00620 32328051 PMC7160646

[33] Posada-Florez F, Lamas ZS, Hawthorne DJ, Chen Y, Evans JD, Ryabov EV. 2021. Pupal cannibalism by worker honey bees contributes to the spread of deformed wing virus. Sci Rep 11:8989. doi:10.1038/s41598-021-88649-y 33903723 PMC8076318

[34] Evans JD, Spivak M. 2010. Socialized medicine: individual and communal disease barriers in honey bees. J Invertebr Pathol 103 Suppl 1:S62–72. doi:10.1016/j.jip.2009.06.019 19909975

[35] Piou V, Schurr F, Dubois E, Vétillard A. 2022. Transmission of deformed wing virus between Varroa destructor foundresses, mite offspring and infested honey bees. Parasites Vectors 15:333. doi:10.1186/s13071-022-05463-9 36151583 PMC9502634

[36] Gisder S, Genersch E. 2021. Direct evidence for infection of Varroa destructor mites with the bee-pathogenic deformed wing virus variant B, but not variant A, via fluorescence in situ hybridization analysis. J Virol 95:e01786-20. doi:10.1128/JVI.01786-20 33298545 PMC8092827

[37] Gusachenko ON, Woodford L, Balbirnie-Cumming K, Campbell EM, Christie CR, Bowman AS, Evans DJ. 2020. Green bees: reverse genetic analysis of deformed wing virus transmission, replication, and tropism. Viruses 12:532. doi:10.3390/v12050532 32408550 PMC7291132

[38] Ongus JR, Peters D, Bonmatin J-M, Bengsch E, Vlak JM, van Oers MM. 2004. Complete sequence of a picorna-like virus of the genus iflavirus replicating in the mite Varroa destructor. J Gen Virol 85:3747–3755. doi:10.1099/vir.0.80470-0 15557248

[39] Eliash N, Suenaga M, Mikheyev AS. 2022. Vector-virus interaction affects viral loads and co-occurrence. BMC Biol. 20:284. doi:10.1186/s12915-022-01463-4 36527054 PMC9758805

[40] Posada-Florez F, Childers AK, Heerman MC, Egekwu NI, Cook SC, Chen Y, Evans JD, Ryabov EV. 2019. Deformed wing virus type A, a major honey bee pathogen, is vectored by the mite Varroa destructor in a non-Propagative manner. Sci Rep 9:12445. doi:10.1038/s41598-019-47447-3 31455863 PMC6712216

[41] Holmes EC. 2009. The evolution and emergence of RNA viruses. Oxford University Press, Oxford.

[42] Herrero S, Millán-Leiva A, Coll S, González-Martínez RM, Parenti S, González-Cabrera J. 2019. Identification of new viral variants specific to the honey bee mite Varroa destructor. Exp Appl Acarol 79:157–168. doi:10.1007/s10493-019-00425-w 31624979

[43] Levin S, Sela N, Chejanovsky N. 2016. Two novel viruses associated with the Apis mellifera pathogenic mite Varroa destructor. Sci Rep 6:37710. doi:10.1038/srep37710 27883042 PMC5121581

[44] Levin S, Sela N, Erez T, Nestel D, Pettis J, Neumann P, Chejanovsky N. 2019. New viruses from the ectoparasite mite Varroa destructor infesting Apis mellifera and Apis cerana. Viruses 11:94. doi:10.3390/v11020094 30678330 PMC6409542

[45] Li N, Li C, Hu T, Li J, Zhou H, Ji J, Wu J, Kang W, Holmes EC, Shi W, Xu S. 2023. Nationwide genomic surveillance reveals the prevalence and evolution of honeybee viruses in China. Microbiome 11:6. doi:10.1186/s40168-022-01446-1 36631833 PMC9832778

[46] de Miranda JR, Cornman RS, Evans JD, Semberg E, Haddad N, Neumann P, Gauthier L. 2015. Genome characterization, prevalence and distribution of a macula-like virus from Apis mellifera and Varroa destructor. Viruses 7:3586–3602. doi:10.3390/v7072789 26154017 PMC4517118

[47] Gauthier L, Cornman S, Hartmann U, Cousserans F, Evans J, de Miranda J, Neumann P. 2015. The Apis mellifera filamentous virus genome. Viruses 7:3798–3815. doi:10.3390/v7072798 26184284 PMC4517127

[48] Cornman SR, Schatz MC, Johnston SJ, Chen Y-P, Pettis J, Hunt G, Bourgeois L, Elsik C, Anderson D, Grozinger CM, Evans JD. 2010. Genomic survey of the ectoparasitic mite Varroa destructor, a major pest of the honey bee Apis mellifera. BMC Genomics 11:602. doi:10.1186/1471-2164-11-602 20973996 PMC3091747

[49] Kraberger S, Visnovsky GA, van Toor RF, Male MF, Waits K, Fontenele RS, Varsani A. 2018. Genome sequences of two single-stranded DNA viruses identified in Varroa destructor . Genome Announc 6. doi:10.1128/genomeA.00107-18 PMC5834324 29496833

[50] Gisder S, Aumeier P, Genersch E. 2009. Deformed wing virus: replication and viral load in mites (Varroa destructor). J Gen Virol 90:463–467. doi:10.1099/vir.0.005579-0 19141457

[51] Liu S, Vijayendran D, Bonning BC. 2011. Next generation sequencing technologies for insect virus discovery. Viruses 3:1849–1869. doi:10.3390/v3101849 22069519 PMC3205385

[52] Kingsolver MB, Huang Z, Hardy RW. 2013. Insect antiviral innate immunity: Pathways, effectors, and connections. J Mol Biol 425:4921–4936. doi:10.1016/j.jmb.2013.10.006 24120681 PMC4007215

[53] Jinek M, Doudna JA. 2009. A three-dimensional view of the molecular machinery of RNA interference. Nature 457:405–412. doi:10.1038/nature07755 19158786

[54] Gammon DB, Mello CC. 2015. RNA interference-mediated antiviral defense in insects. Curr Opin Insect Sci 8:111–120. doi:10.1016/j.cois.2015.01.006 26034705 PMC4448697

[55] Webster CL, Waldron FM, Robertson S, Crowson D, Ferrari G, Quintana JF, Brouqui J-M, Bayne EH, Longdon B, Buck AH, Lazzaro BP, Akorli J, Haddrill PR, Obbard DJ. 2015. The discovery, distribution, and evolution of viruses associated with Drosophila melanogaster. PLoS Biol 13:e1002210. doi:10.1371/journal.pbio.1002210 26172158 PMC4501690

[56] Chejanovsky N, Ophir R, Schwager MS, Slabezki Y, Grossman S, Cox-Foster D. 2014. Characterization of viral siRNA populations in honey bee colony collapse disorder. Virology 454–455:176–183. doi:10.1016/j.virol.2014.02.012 24725944

[57] Santos D, Mingels L, Vogel E, Wang L, Christiaens O, Cappelle K, Wynant N, Gansemans Y, Van Nieuwerburgh F, Smagghe G, Swevers L, Vanden Broeck J. 2019. Generation of Virus- and dsRNA-derived siRNAs with species-dependent length in insects. Viruses 11:738. doi:10.3390/v11080738 31405199 PMC6723321

[58] Remnant EJ, Shi M, Buchmann G, Blacquière T, Holmes EC, Beekman M, Ashe A. 2017. A diverse range of novel RNA viruses in geographically distinct honey bee populations. J Virol 91:e00158-17. doi:10.1128/JVI.00158-17 28515299 PMC5533899

[59] Nganso BT, Sela N, Soroker V. 2020. A genome-wide screening for RNAi pathway proteins in Acari. BMC Genomics 21:791. doi:10.1186/s12864-020-07162-0 33183236 PMC7659050

[60] Maxwell CC, Carolyn NE, Michael B, Michael BE. 2018. Entomophthovirus: an insect-derived Iflavirus that Infects a behavior manipulating fungal pathogen of Dipterans. bioRxiv. doi:10.1101/371526:371526 PMC11457076 39158097

[61] Kumar D, Alburaki M, Tahir F, Goblirsch M, Adamczyk J, Karim S. 2022. An insight into the microRNA profile of the ectoparasitic mite Varroa destructor (Acari: Varroidae), the primary vector of honey bee deformed wing virus. Front Cell Infect Microbiol 12:847000. doi:10.3389/fcimb.2022.847000 35372101 PMC8966896

[62] Krueger F, Trimgalore. 2021. Github repository. Available from: 10.5281/zenodo.5127898

[63] Andrews S. 2010. Online. FastQC: a quality control tool for high throughput sequence data. Available from: http://www.bioinformatics.babraham.ac.uk/projects/fastqc/

[64] Langmead B, Salzberg SL. 2012. Fast Gapped-read alignment with Bowtie 2. Nat Meth 9:357–359. 10.1038/nmeth.1923 PMC3322381 22388286

[65] Li H, Handsaker B, Wysoker A, Fennell T, Ruan J, Homer N, Marth G, Abecasis G, Durbin R. 2009. The sequence alignment/map format and Samtools. Bioinformatics 25:2078–2079. doi:10.1093/bioinformatics/btp352 19505943 PMC2723002

[66] Li D, Liu C-M, Luo R, Sadakane K, Lam T-W. 2015. MEGAHIT: an ultra-fast single-node solution for large and complex metagenomics assembly via succinct de Bruijn graph. Bioinformatics 31:1674–1676. doi:10.1093/bioinformatics/btv033 25609793

[67] Charon J, Buchmann JP, Sadiq S, Holmes EC. 2022. RdRp-scan: a bioinformatic resource to identify and annotate divergent RNA viruses in metagenomic sequence data. Virus Evol 8:veac082. doi:10.1093/ve/veac082 36533143 PMC9752661

[68] Eddy SR, Pearson WR. 2011. Accelerated profile HMM searches. PLOS Comput Biol 7:e1002195. doi:10.1371/journal.pcbi.1002195 22039361 PMC3197634

[69] Edgar RC. 2004. MUSCLE: Multiple sequence alignment with high accuracy and high throughput. Nucleic Acids Research 32:1792–1797. doi:10.1093/nar/gkh340 15034147 PMC390337

[70] Nguyen L-T, Schmidt HA, von Haeseler A, Minh BQ. 2015. IQ-TREE: a fast and effective stochastic algorithm for estimating maximum-likelihood phylogenies. Mol Biol Evol 32:268–274. doi:10.1093/molbev/msu300 25371430 PMC4271533

[71] Kalyaanamoorthy S, Minh BQ, Wong TKF, von Haeseler A, Jermiin LS. 2017. ModelFinder: fast model selection for accurate phylogenetic estimates. Nat Methods 14:587–589. doi:10.1038/nmeth.4285 28481363 PMC5453245

[72] Hoang DT, Chernomor O, von Haeseler A, Minh BQ, Vinh LS. 2018. UFBoot2: improving the Ultrafast Bootstrap approximation. Mol Biol Evol 35:518–522. doi:10.1093/molbev/msx281 29077904 PMC5850222

[73] Shi M, Lin X-D, Tian J-H, Chen L-J, Chen X, Li C-X, Qin X-C, Li J, Cao J-P, Eden J-S, Buchmann J, Wang W, Xu J, Holmes EC, Zhang Y-Z. 2016. Redefining the Invertebrate RNA virosphere. Nature 540:539–543. doi:10.1038/nature20167 27880757

[74] Koonin EV, Dolja VV, Krupovic M, Varsani A, Wolf YI, Yutin N, Zerbini FM, Kuhn JH. 2020. Global organization and proposed megataxonomy of the virus world. Microbiol Mol Biol Rev 84:e00061–19. doi:10.1128/MMBR.00061-19 32132243 PMC7062200

[75] Schuster S, Zirkel F, Kurth A, van Cleef KWR, Drosten C, van Rij RP, Junglen S. 2014. A unique nodavirus with novel features: mosinovirus expresses two subgenomic RNAs, a capsid gene of unknown origin, and a suppressor of the antiviral RNA interference pathway. J Virol 88:13447–13459. doi:10.1128/JVI.02144-14 25210176 PMC4249075

[76] Ashe A, Bélicard T, Le Pen J, Sarkies P, Frézal L, Lehrbach NJ, Félix M-A, Miska EA. 2013. A deletion polymorphism in the Caenorhabditis elegans RIG-I homolog disables viral RNA Dicing and antiviral immunity. Elife 2:e00994. doi:10.7554/eLife.00994 24137537 PMC3793227

[77] Brackney DE, Beane JE, Ebel GD. 2009. RNAi targeting of West Nile virus in mosquito midguts promotes virus diversification. PLoS Pathog 5:e1000502. doi:10.1371/journal.ppat.1000502 19578437 PMC2698148

[78] Waldron FM, Stone GN, Obbard DJ. 2018. Metagenomic sequencing suggests a diversity of RNA interference-like responses to viruses across multicellular eukaryotes. PLoS Genet 14:e1007533. doi:10.1371/journal.pgen.1007533 30059538 PMC6085071

[79] Sekhar Nandety R, Fofanov VY, Koshinsky H, Stenger DC, Falk BW. 2013. Small RNA populations for two unrelated viruses exhibit different biases in strand polarity and proximity to terminal sequences in the insect host Homalodisca vitripennis. Virology 442:12–19. doi:10.1016/j.virol.2013.04.005 23642540

[80] Levin S, Galbraith D, Sela N, Erez T, Grozinger CM, Chejanovsky N. 2017. Presence of Apis Rhabdovirus-1 in populations of Pollinators and their parasites from two continents. Front Microbiol 8:2482. doi:10.3389/fmicb.2017.02482 29312191 PMC5732965

[81] Kadlečková D, Tachezy R, Erban T, Deboutte W, Nunvář J, Saláková M, Matthijnssens J. 2022. The Virome of healthy honey bee colonies: ubiquitous occurrence of known and new viruses in bee populations. mSystems 7:e0007222. doi:10.1128/msystems.00072-22 35532210 PMC9239248

[82] Roberts JMK, Simbiken N, Dale C, Armstrong J, Anderson DL. 2020. Tolerance of honey bees to Varroa mite in the absence of deformed wing virus. Viruses 12:575. doi:10.3390/v12050575 32456246 PMC7290856

[83] Obbard DJ, Shi M, Roberts KE, Longdon B, Dennis AB. 2020. A new lineage of segmented RNA viruses infecting animals. Virus Evol 6:vez061. doi:10.1093/ve/vez061 31976084 PMC6966834

[84] McDonald SM, Nelson MI, Turner PE, Patton JT. 2016. Reassortment in segmented RNA viruses: mechanisms and outcomes. Nat Rev Microbiol 14:448–460. doi:10.1038/nrmicro.2016.46 27211789 PMC5119462

[85] Bailey L. 1967. The incidence of virus diseases in the honey bee. Ann Appl Biol 60:43–48. doi:10.1111/j.1744-7348.1967.tb05920.x 6076167

[86] Roberts JMK, Anderson DL, Durr PA. 2017. Absence of deformed wing virus and Varroa destructor in Australia provides unique perspectives on honeybee viral landscapes and colony losses. Sci Rep 7:6925. doi:10.1038/s41598-017-07290-w 28761114 PMC5537221

[87] Tentcheva D, Gauthier L, Zappulla N, Dainat B, Cousserans F, Colin ME, Bergoin M. 2004. Prevalence and seasonal variations of six bee viruses in Apis mellifera L and Varroa destructor mite populations in France. Appl Environ Microbiol 70:7185–7191. doi:10.1128/AEM.70.12.7185-7191.2004 15574916 PMC535170

[88] Sabahi Q, Morfin N, Nehzati-Paghaleh G, Guzman-Novoa E. 2020. Detection and replication of deformed wing virus and black Queen cell virus in parasitic mites, Varroa destructor, from Iranian honey bee (Apis Mellifera) colonies . Journal of Apicultural Research 59:211–217. doi:10.1080/00218839.2019.1686576

[89] Bailey L. 1969. The multiplication and spread of sacbrood virus of bees. Ann Appl Biol 63:483–491. doi:10.1111/j.1744-7348.1969.tb02844.x 5806364

[90] Bailey L, Woods RD. 1977. Two more small RNA viruses from honey bees and further observations on sacbrood and acute bee-paralysis viruses. J Gen Vir 37:175–182. doi:10.1099/0022-1317-37-1-175

[91] Remnant EJ, Mather N, Gillard TL, Yagound B, Beekman M. 2019. Direct transmission by injection affects competition among RNA viruses in honeybees. Proc Biol Sci 286:20182452. doi:10.1098/rspb.2018.2452 30963951 PMC6364586

[92] Martin SJ. 2001. The role of Varroa and viral pathogens in the collapse of honeybee colonies: a modelling approach. J of App Eco 38:1082–1093. doi:10.1046/j.1365-2664.2001.00662.x

[93] Beaurepaire A, Piot N, Doublet V, Antunez K, Campbell E, Chantawannakul P, Chejanovsky N, Gajda A, Heerman M, Panziera D, Smagghe G, Yañez O, de Miranda JR, Dalmon A. 2020. Diversity and global distribution of viruses of the Western honey bee, Apis mellifera. Insects 11:239. doi:10.3390/insects11040239 32290327 PMC7240362

[94] Ryabov EV, Posada-Florez F, Rogers C, Lamas ZS, Evans JD, Chen Y, Cook SC. 2022. The vectoring competence of the mite Varroa destructor for deformed wing virus of honey bees is dynamic and affects survival of the mite. Front Insect Sci 2. doi:10.3389/finsc.2022.931352 PMC10926515 38468796

[95] Akay A, Sarkies P, Miska EA. 2015. E. coli OxyS non-coding RNA does not trigger RNAi in C. elegans. Sci Rep 5:9597. doi:10.1038/srep09597 25873159 PMC4397834

[96] Pinzón N, Bertrand S, Subirana L, Busseau I, Escrivá H, Seitz H. 2019. Functional Lability of RNA-dependent RNA polymerases in animals. PLOS Genet. 15:e1007915. doi:10.1371/journal.pgen.1007915 30779744 PMC6396948

[97] Schnettler E, Tykalová H, Watson M, Sharma M, Sterken MG, Obbard DJ, Lewis SH, McFarlane M, Bell-Sakyi L, Barry G, Weisheit S, Best SM, Kuhn RJ, Pijlman GP, Chase-Topping ME, Gould EA, Grubhoffer L, Fazakerley JK, Kohl A. 2014. Induction and suppression of tick cell antiviral RNAi responses by tick-borne flaviviruses. Nucleic Acids Res. 42:9436–9446. doi:10.1093/nar/gku657 25053841 PMC4132761

[98] Waterhouse PM, Fusaro AF. 2006. Viruses face a double defense by plant small Rnas. Science 313:54–55. doi:10.1126/science.1130818 16825558

[99] Borges F, Martienssen RA. 2015. The expanding world of small RNAs in plants. Nat Rev Mol Cell Biol 16:727–741. doi:10.1038/nrm4085 26530390 PMC4948178

