# Supplementary material for: Virus replication in the honey bee parasite, Varroa destructor
Source: J Virol. 2023 Nov 15;97(12):e01149-23. doi: 10.1128/jvi.01149-23 (PMC10746231; doi:10.1128/jvi.01149-23)
Supplement: Supplemental tables and figures — Tables S1, S2 and Figures S1, S2, S3, S4, S5. [file jvi.01149-23-s0001.pdf]

Supplementary tables and Figures

Table S1. *Varroa destructor* small RNA samples used in this study.

Table S2. *Varroa destructor* associated virus genomes used to generate virus reference library.

Figure S1. Comparison of the proportion of small RNA reads mapping to virus genomes before and after consensus sequence generation

Figure S2. Phylogenetic analysis of honey bee-associated viruses in *V. destructor* samples.

Figure S3. Phylogenetic analysis of consensus sequences of *V. destructor*-associated viruses.

Figure S4. Additional examples of size profiles of viral small RNA reads mapping to viruses

Figure S5. Coverage plots of antisense viral small RNA reads mapping to DWV

Supplementary file 1. Spreadsheet showing the number and proportion of sense and antisense reads mapping to the viruses identified in this study:

- ARV-1
- ARV-2
- VDV-2
- VDV-3
- VDV-5
- VDV-9
- DWV-A
- DWV-B
- BMLV
- BQCV
- SBV

Table S1. *Varroa destructor* small RNA samples used in this study. All samples are individual whole mites other than those denoted with p, which consist of pooled mites. In our samples we pooled 4 mites, while other studies pooled 20 (CH-1p, CH-2p, CH-3p, US-1p) or 10 mites (US-2p).

Sample	Location	Collection date	Biosample ID (SRA)	Reference
NE-1	Wageningen, Netherlands	Aug 2018	SRR25010770	*This study*
NE-2	Wageningen, Netherlands	Aug 2018	SRR25010769	*This study*
NE-3	Wageningen, Netherlands	Aug 2018	SRR25010758	*This study*
NE-4	Wageningen, Netherlands	Sep 2018	SRR25010756	*This study*
NE-5	Wageningen, Netherlands	Sep 2018	SRR25010755	*This study*
NE-6	Wageningen, Netherlands	Sep 2018	SRR25010754	*This study*
NE-7p	Wageningen, Netherlands	Aug 2018	SRR25010753	*This study*
NE-8p	Wageningen, Netherlands	Aug 2018	SRR25010752	*This study*
NZ-1	Wellington, New Zealand	Mar 2022	SRR25010751	*This study*
NZ-2	Wellington, New Zealand	Mar 2022	SRR25010750	*This study*
NZ-3	Wellington, New Zealand	Mar 2022	SRR25010768	*This study*
NZ-4	Wellington, New Zealand	Mar 2022	SRR25010767	*This study*
NZ-5	Hamilton, New Zealand	Mar 2015	SRR25010766	*This study*
NZ-6	Wellington, New Zealand	Mar 2022	SRR25010765	*This study*
NZ-7	Wellington, New Zealand	Mar 2022	SRR25010764	*This study*
NZ-8p	Wellington, New Zealand	Mar 2022	SRR25010763	*This study*
NZ-9p	Wellington, New Zealand	Mar 2022	SRR25010762	*This study*
SA-1	Robben Island, South Africa	Mar 2013	SRR5109825	(1)
SA-2	Robben Island, South Africa	Mar 2013	SRR5109827	(1)
SA-3	Kromme Rhee, South Africa	Nov 2016	SRR25010761	*This study*
SA-4	Kromme Rhee, South Africa	Nov 2016	SRR25010760	*This study*
SA-5p	Elsenburg, South Africa	Nov 2016	SRR25010759	*This study*
SA-6p	Elsenburg, South Africa	Nov 2016	SRR25010757	*This study*
CH-1p	Jilin, China	Oct 2018	SRR13959567	(2)
CH-2p	Heilongjian, China	Oct 2018	SRR13959568	(2)
CH-3p	Hebei, China	Nov 2018	SRR13959569	(2)
US-1p	Mississippi, USA	Oct 2019	SRR17429192	(3)
US-2p	Mississippi, USA	Oct 2019	SRR17429196	(3)

References:

1. Remnant EJ, Shi M, Buchmann G, Blaquiere T, Holmes EC, Beekman M, Ashe A. 2017. A diverse range of novel RNA viruses in geographically distinct honey bee populations. J Virol 91.

2. Li N, Li C, Hu T, Li J, Zhou H, Ji J, Wu J, Kang W, Holmes EC, Shi W, Xu S. 2023. Nationwide genomic surveillance reveals the prevalence and evolution of honeybee viruses in China. Microbiome 11:6.

3. Kumar D, Alburaki M, Tahir F, Goblirsch M, Adamczyk J, Karim S. 2022. An Insight Into the microRNA Profile of the Ectoparasitic Mite Varroa destructor (Acari: Varroidae), the Primary Vector of Honey Bee Deformed Wing Virus. 12.

Table S2: Viral reference library containing *V. destructor* virus species previously reported in (1, 2), as well as viruses identified in this study, and other pathogenic bee viruses.

Acronym	Virus	Accession
AACV	Anguilla anguilla circovirus	NC_023421.1
ABPV	Acute bee paralysis virus	NC_002548.1
ABPV	Acute bee paralysis virus	AF150629.1
ABV	Aransas bay virus	KC506163.1
ADCV-2	Apis dicistrovirus 2	MZ822070.1
ADCV-3	Apis dicistrovirus 3	MZ822071.1
ADCV-4	Apis dicistrovirus 4	MZ822072.1
AHV-1	Apis hypovirus 1	MZ822101.1
AHV-2	Apis hypovirus 2	MZ822102.1
AHV-3	Apis hypovirus 3	MZ822103.1
AIV-1	Apis iflavirus 1	MZ822075.1
AIV-2	Apis iflavirus 2	MZ822076.1
AmFv	Apis mellifera filamentous virus	NC_027925.1
ANV-2	Apis nora virus 2	MZ822097.1
APCV	Apis polycipivirus	MZ822098.1
APLV-1	Apis picorna-like virus 1	MZ822067.1
APLV-2	Apis picorna-like virus 2	MZ822068.1
APLV-3	Apis picorna-like virus 3	MZ822078.1
APLV-4	Apis picorna-like virus 4	MZ822093.1
APLV-4	Apis picorna-like virus 4	MZ822094.1
APLV-5	Apis picorna-like virus 5	MZ822095.1
APLV-6	Apis picorna-like virus 6	MZ822096.1
ARV-1	Apis rhabdovirus 1	MF114351.1
ARV-2	Apis rhabdovirus 2	MZ821796.1
ARV-3	Apis rhabdovirus 3	MZ822104.1
ARV-4	Apis rhabdovirus 4	MZ822105.1
ARV-5	Apis rhabdovirus 5	MZ822106.1
AVD	Antheraea pernyi iflavirus	KF751885.1
AVLB	Apis virga-like virus	MZ822100.1
BBV-1	Bundaberg bee virus 1	MG995701.1
BCVZS	Bat circovirus ZS	JF938080.1
BfaPLV	Bat feces associated picorna-like virus	JN857331.1
BHCV-1	Beihai horseshoe crab virus 1	KX883115.1
BMLV	Bee Macula-like virus (Netherlands isolates, this study)	OR224322, OR224323, OR224324
BMLV	Bee Macula-like virus	KT162924.1
BQCV	Black queen cell virus	NC_003784.1
CBPV	Chronic bee paralysis virus	NC_010711.1
CBPV	Chronic bee paralysis virus	EU122230.1
CV-NG14	Cyclovirus NG14	GQ404855.1
CV-NGCh8	Cyclovirus NGchicken8	HQ738643.1
CV-TN18	Cyclovirus TN18	GQ404858.1
CV-TN25	Cyclovirus TN25	GQ404857.1
DfCV-1	Dragonfly cyclovirus 1	JX185421.1
DfCV-4	Dragonfly cyclovirus 4	KC512917.1
DfCV-6	Dragonfly cyclovirus 6	KC512918.1
DHOV	Dhori virus concat	NC_034255.1
DWV-A	Deformed wing virus A	AY292384.1
DWV-A_NZ	Deformed wing virus A NZ isolate	MN538208.1
DWV-B	Varroa destructor virus 1	AY251269.2
DWV-B_NE	Deformed wing virus B Netherlands isolate	MN538209.1
ELV	Erysimum latent virus	AF098523.1
EppoNPV	Epiphyas postvittana NPV	NC_003083.1
FARV	Farmington virus	HM627182.1
FeV-1	Formica exsecta virus 1	KF500001.1
FeV-2	Formica exsecta virus 2	KF500002.1
GRGV	Grapevine Red Globe virus	LC704878.1
HelV	Heliconius erato iflavirus	KJ679438.1
HhV	Halyomorpha halys virus	KF699344.1
HPLV-22	Hubei picorna-like virus 22	NC_033227.1
HPLV-29	Hubei picorna-like virus 29	KX883292.1
IAPV	Israel acute paralysis virus	EF219380.1

Acronym	Virus	Accession
IAPV	Israel acute paralysis virus	EU375541.1
JFV-2	Jingshan Fly Virus 2	KM817631.1
JOSV	Jos virus	HM627170.1
KBV	Kashmir bee virus	AY275710.1
LsPV-2	Laodelphax striatellus picorna-like virus 2	KM272628.1
LSV-1	Lake Sinai virus 1	HQ871931.2
LSV-1	Lake Sinai virus 1	MT482469.1
LSV-2	Lake Sinai virus 2	HQ888865.2
LSV-2	Lake Sinai virus 2	MT482482.1
LSV-3	Lake Sinai virus 3	MH267700.1
LSV-3	Lake Sinai virus 3	MT482473.1
LSV-8	Lake Sinai virus 8	MT482470.1
MoNV	Mosinovirus strain	KJ632942.1
PhMV	Physalis mottle virus	NC_003634.1
PlMV	Plantago mottle virus	AY751779.1
PLVEf	Picorna-like virus Eptesicus fuscus	HQ585111.1
SBPV	Slow bee paralysis virus	EU035616.1
SBPV	Slow bee paralysis virus	NC_014137.1
SBV	Sacbrood virus	AF092924.1
SCRMV	Scrophularia mottle virus	AY751777.1
SeIV-1	Spodoptera exigua Iflavirus-1	JN091707.1
THOV	Thogoto virus	NC_006504.1
TLV	Tjuloc virus	JQ928941.1
ToBMV	Tomato blistering mosaic virus	KJ940970.1
TpIV-1	Thaumetopoea pityocampa iflavirus 1	KP217032.1
TYMV	Turnip yellow mosaic virus	NC_004063.1
VDCV-1	Varroa dicistrovirus 1	MZ822069.1
VDCV-2	Varroa dicistrovirus 2	MZ822073.1
VdMLV	Varroa destructor Macula-like virus	AB859949.1
VDV-2_China	Varroa destructor virus 2, China isolate	MW590582.1
VDV-2_Israel	Varroa destructor virus 2, Israel isolate	KX578271.1
VDV-2_UK	Varroa destructor virus 2, UK isolate	MK795517.1
VDV-3	Varroa destructor virus 3	KX578272.1
VDV-3_NE	Varroa destructor virus 3, Netherlands isolate	*unpublished*
VDV-5	Varroa destructor virus 5	MK795519.1
VDV-5_NZ	Varroa destructor virus 5, NZ isolate	*unpublished*
VDV-9_NE	Varroa destructor virus 9, Netherlands isolate (this study)	OR224321
VDV-9_NZ	Varroa destructor virus 9, NZ isolate (this study)	OR224325
VOV-1	Varroa orthomyxovirus 1	OL803864.1
VPLV	Varroa picorna-like virus	MZ822077.1
WFBV	Wellfeet Bay virus	NC_025793.1
WWVV	Watercress white vein virus	JQ001816.1

References:

1. Lester PJ, Felden A, Baty JW, Bulgarella M, Haywood J, Mortensen AN, Remnant EJ, Smeele ZE. 2022. Viral communities in the parasite Varroa destructor and in colonies of their honey bee host (*Apis mellifera*) in New Zealand. Scientific Reports 12:8809.
2. Li N, Li C, Hu T, Li J, Zhou H, Ji J, Wu J, Kang W, Holmes EC, Shi W, Xu S. 2023. Nationwide genomic surveillance reveals the prevalence and evolution of honeybee viruses in China. Microbiome 11:6.

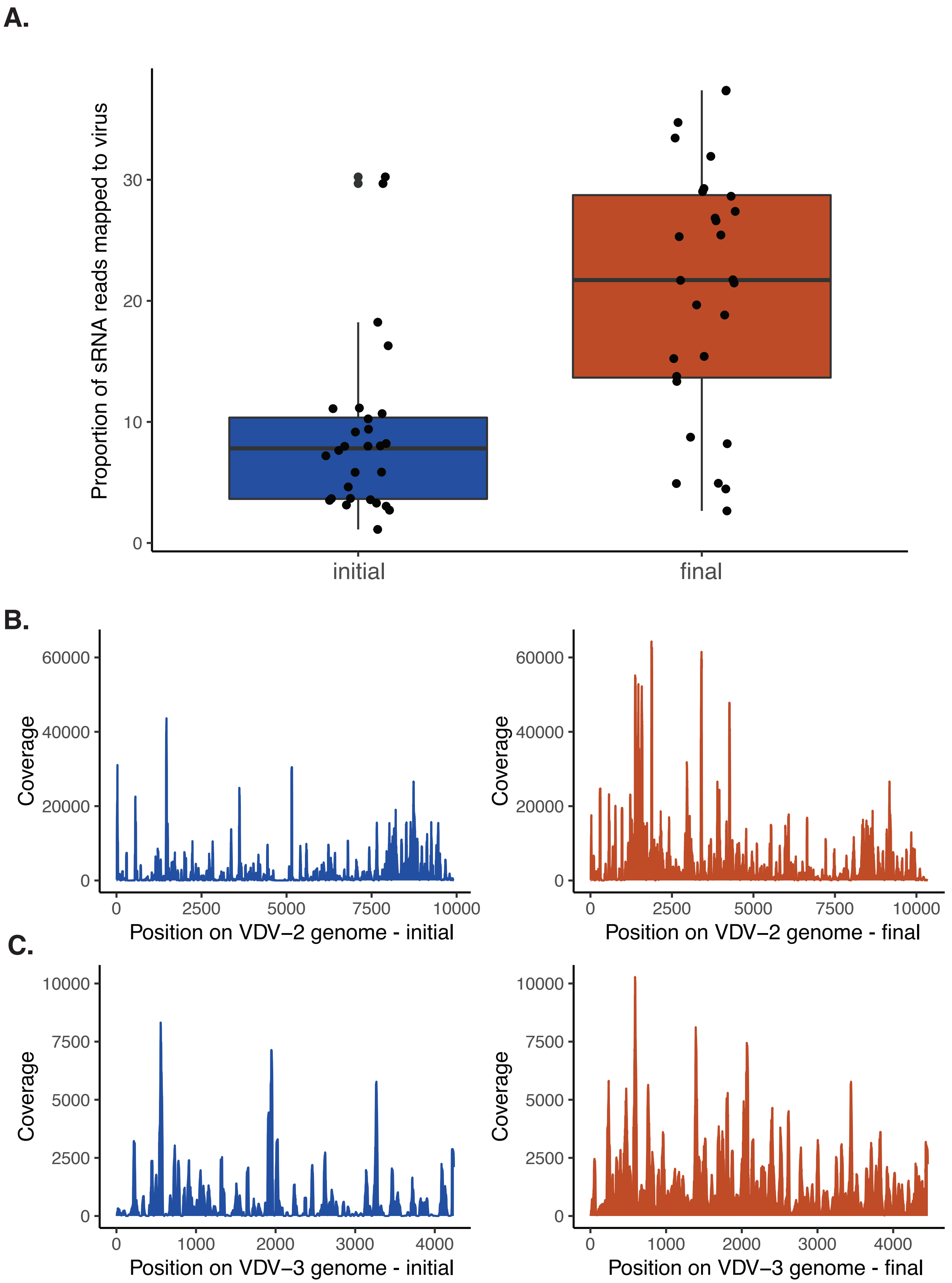

Figure S1. A) Comparison of the proportion of small RNA reads mapping to reference virus genomes (initial; blue) versus the proportion of reads mapping to consensus genomes generated for each virus after iterative mapping (final; orange). **B-C)** Coverage plots of **B)**VDV-2 and **C)** VDV-3 genomes of reads mapping to virus reference genomes (initial; blue) versus coverage of reads mapping to consensus genomes generated after iterative mapping (final; orange), showing improved coverage and reduced regions with gaps. Representative samples are shown (**B)** VDV-2: NZ-3; **C)** VDV-3: SA_5p).

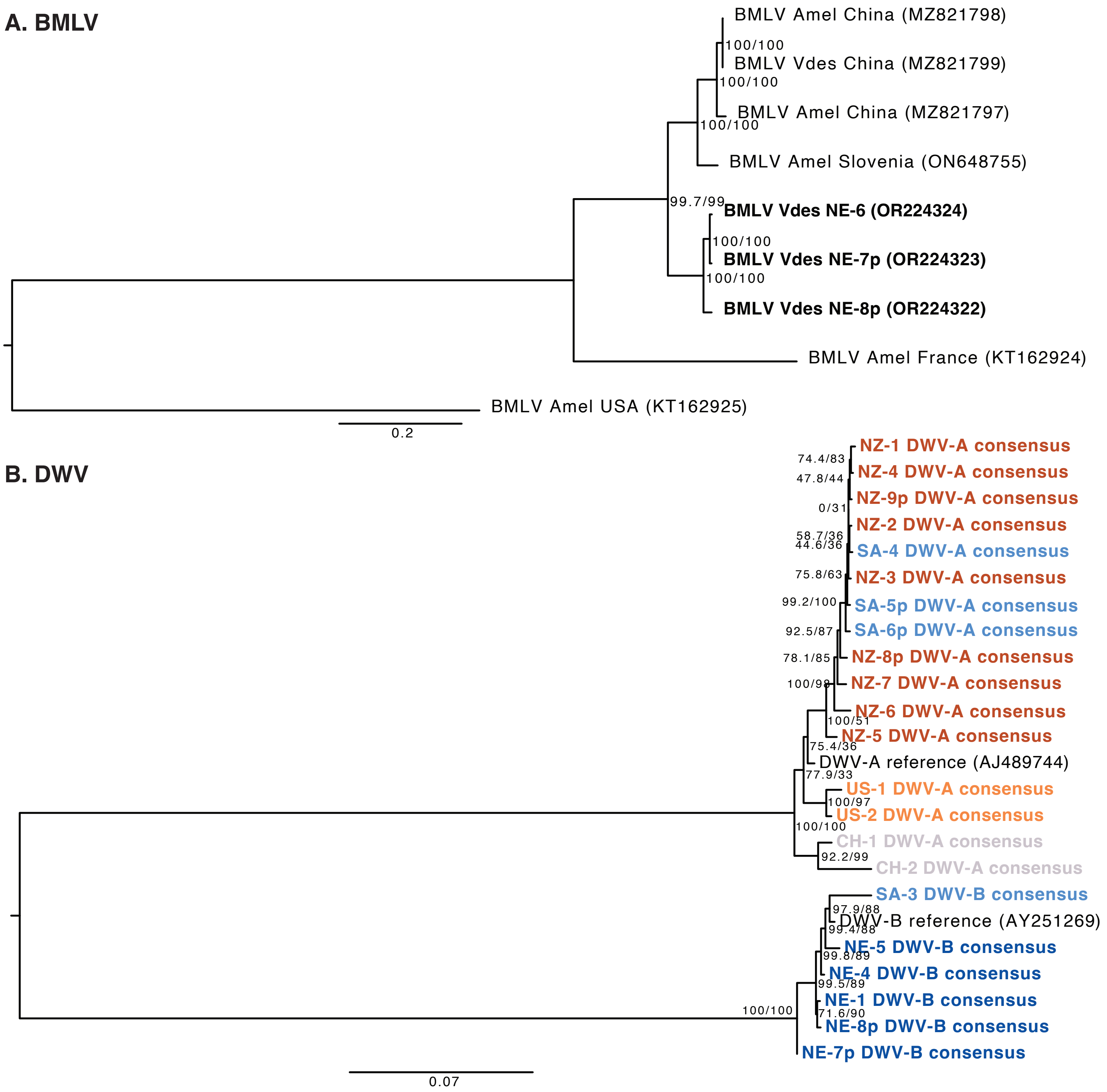

Figure S2. Phylogenetic analysis of honey bee- associated viruses in *V. destructor* samples. **A)** Bee macula-like virus isolates from three *V. destructor* samples from the Netherlands (OR224322-OR224324). Whole genome nucleotide sequence alignments were aligned using MUSCLE. After trimming, alignment length was 5930 nt. The phylogenetic tree was generated using maximum likelihood in IQ-TREE [1], with the TN+F+I+G4 model which had the optimal BIC score, as determined by ModelFinder [2]. **B)** Deformed wing virus consensus sequences from positive *V. destructor* samples. Whole genome nucleotide sequences were aligned as above and samples with evidence of recombination were removed. The final length after trimming was 9198 nt. Phylogenetic tree generation and model prediction were performed as above, with the TN+F+G4 model chosen according to BIC. For both A and B, branch supports were estimated using Ultrafast bootstrap approximation (UFBoot; [3]) using 1000 replicates. Support values shown are SH-aLRT and UFBoot supports.

1. Nguyen, L.-T.; Schmidt, H.A.; von Haeseler, A.; Minh, B.Q. Iq-tree: A fast and effective stochastic algorithm for estimating maximum-likelihood phylogenies. Mol Biol Evol 2014, 32, 268-274.
2. Kalyaanamoorthy, S.; Minh, B.Q.; Wong, T.K.F.; von Haeseler, A.; Jermiin, L.S. Modelfinder: Fast model selection for accurate phylogenetic estimates. Nat Methods 2017, 14.
3. Hoang, D.T.; Chernomor, O.; von Haeseler, A.; Minh, B.Q.; Vinh, L.S. Ufboot2: Improving the ultrafast bootstrap approximation. Mol Biol Evol 2017, 35, 518-522.

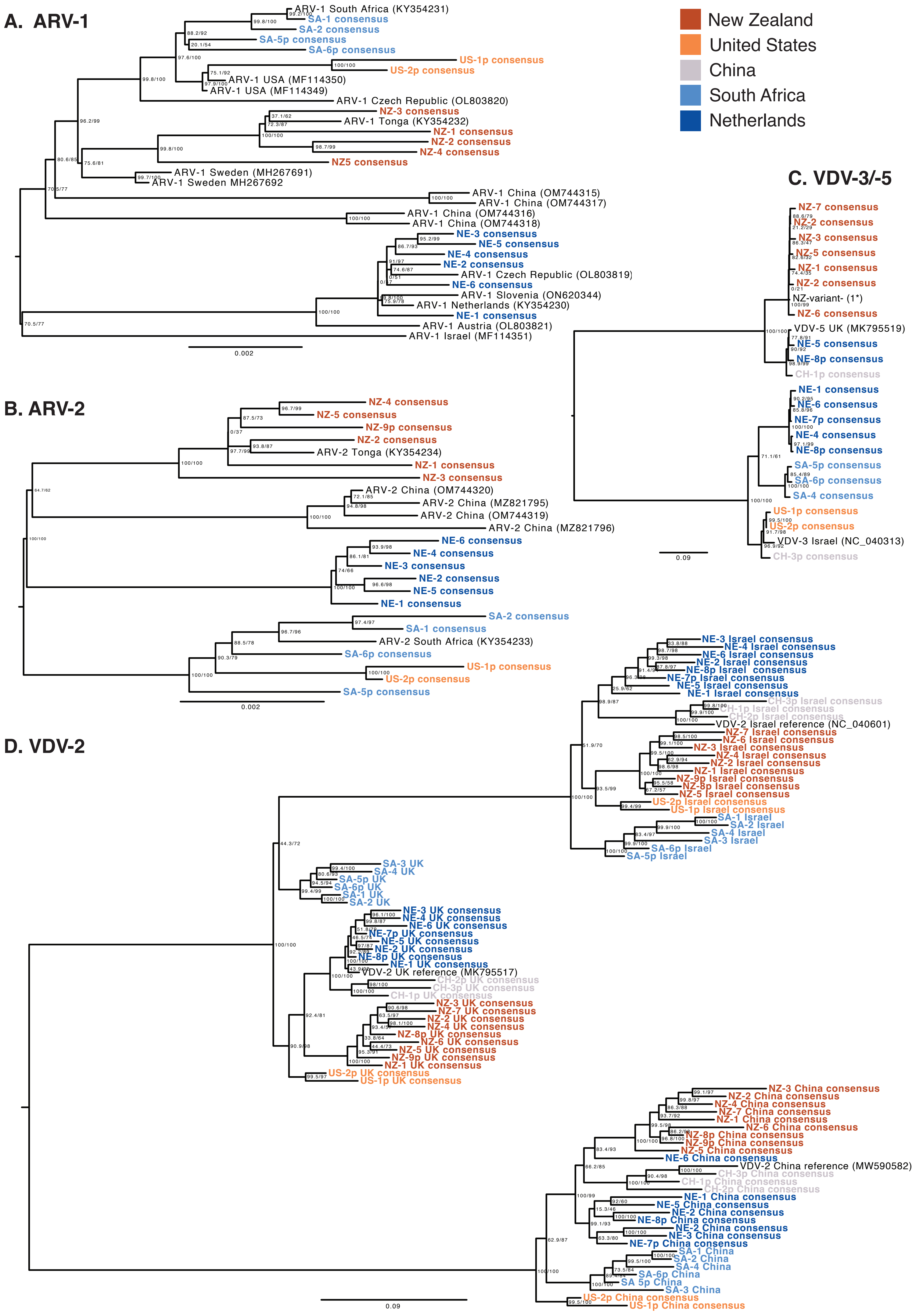

A. ARV-1
ARV-1 South Africa (KY354231)
SA-1 consensus
SA-2 consensus
SA-5p consensus
SA-6p consensus
US-1p consensus
US-2p consensus
ARV-1 USA (MF114350)
ARV-1 USA (MF114349)
ARV-1 Czech Republic (OL803820)
NZ-3 consensus
ARV-1 Tonga (KY354232)
NZ-1 consensus
NZ-2 consensus
NZ-4 consensus
NZ5 consensus
ARV-1 Sweden (MH267691)
ARV-1 Sweden MH267692
ARV-1 China (OM744315)
ARV-1 China (OM744317)
ARV-1 China (OM744316)
ARV-1 China (OM744318)
NE-3 consensus
NE-5 consensus
NE-4 consensus
NE-2 consensus
ARV-1 Czech Republic (OL803819)
NE-6 consensus
ARV-1 Slovenia (ON620344)
ARV-1 Netherlands (KY354230)
NE-1 consensus
ARV-1 Austria (OL803821)
ARV-1 Israel (MF114351)
0.002
New Zealand
United States
China
South Africa
Netherlands
C. VDV-3/-5
NZ-7 consensus
NZ-2 consensus
NZ-3 consensus
NZ-5 consensus
NZ-1 consensus
NZ-2 consensus
NZ-variant- (1*)
NZ-6 consensus
VDV-5 UK (MK795519)
NE-5 consensus
NE-8p consensus
CH-1p consensus
NE-1 consensus
NE-6 consensus
NE-7p consensus
NE-4 consensus
NE-8p consensus
SA-5p consensus
SA-6p consensus
SA-4 consensus
US-1p consensus
US-2p consensus
VDV-3 Israel (NC_040313)
CH-3p consensus
0.09
B. ARV-2
NZ-4 consensus
NZ-5 consensus
NZ-9p consensus
NZ-2 consensus
ARV-2 Tonga (KY354234)
NZ-1 consensus
NZ-3 consensus
ARV-2 China (OM744320)
ARV-2 China (MZ821795)
ARV-2 China (OM744319)
ARV-2 China (MZ821796)
NE-6 consensus
NE-4 consensus
NE-3 consensus
NE-2 consensus
NE-5 consensus
NE-1 consensus
SA-2 consensus
SA-1 consensus
ARV-2 South Africa (KY354233)
SA-6p consensus
US-1p consensus
US-2p consensus
SA-5p consensus
0.002
D. VDV-2
NE-3 Israel consensus
NE-4 Israel consensus
NE-6 Israel consensus
NE-2 Israel consensus
NE-8p Israel consensus
NE-7p Israel consensus
NE-5 Israel consensus
NE-1 Israel consensus
CH-3p Israel consensus
CH-1p Israel consensus
CH-2p Israel consensus
VDV-2 Israel reference (NC_040601)
NZ-7 Israel consensus
NZ-6 Israel consensus
NZ-3 Israel consensus
NZ-4 Israel consensus
NZ-2 Israel consensus
NZ-1 Israel consensus
NZ-9p Israel consensus
NZ-8p Israel consensus
NZ-5 Israel consensus
US-2p Israel consensus
US-1p Israel consensus
SA-1 Israel
SA-2 Israel
SA-4 Israel
SA-3 Israel
SA-6p Israel
SA-5p Israel
SA-3 UK
SA-4 UK
SA-5p UK
SA-6p UK
SA-1 UK
SA-2 UK
NE-3 UK consensus
NE-4 UK consensus
NE-6 UK consensus
NE-7p UK consensus
NE-5 UK consensus
NE-2 UK consensus
NE-8p UK consensus
NE-1 UK consensus
VDV-2 UK reference (MK795517)
CH-2p UK consensus
CH-3p UK consensus
CH-1p UK consensus
NZ-3 UK consensus
NZ-7 UK consensus
NZ-2 UK consensus
NZ-4 UK consensus
NZ-8p UK consensus
NZ-6 UK consensus
NZ-5 UK consensus
NZ-9p UK consensus
NZ-1 UK consensus
US-2p UK consensus
US-1p UK consensus
NZ-3 China consensus
NZ-2 China consensus
NZ-4 China consensus
NZ-7 China consensus
NZ-1 China consensus
NZ-6 China consensus
NZ-8p China consensus
NZ-9p China consensus
NZ-5 China consensus
NE-6 China consensus
VDV-2 China reference (MW590582)
CH-3p China consensus
CH-1p China consensus
CH-2p China consensus
NE-1 China consensus
NE-5 China consensus
NE-2 China consensus
NE-8p China consensus
NE-2 China consensus
NE-3 China consensus
NE-7p China consensus
SA-1 China
SA-2 China
SA-4 China
SA-6p China
SA 5p China
SA-3 China
US-2p China consensus
US-1p China consensus
0.09

Figure S3. Phylogenetic analysis of consensus sequences of *V. destructor- associated* viruses. Alignments were performed using consensus sequences generated from iterative mapping of V. destructor small RNA reads from each sample to **A)** Apis rhabdovirus 1 (ARV-1); **B)** Apis rhabdovirus 2 (ARV-2); **C)** Varroa destructor virus 3 & 5 (VDV-3/-5) and **D)** Varroa destructor virus 2 (VDV-2), using all three available reference strains from the UK (MK795517), Israel (NC_040601) & China (MW590582).
A) Whole genome consensus sequences of ARV-1 were aligned using MUSCLE and trimmed for gaps, leaving a final length of 12835 nt. The phylogenetic tree was generated using maximum likelihood in IQ-TREE [1], with the TIM+F+G4 model which had the optimal BIC score, as determined by ModelFinder [2]. **B)** ARV-2 was analysed as above, with final alignment length of 12718 nt and the TIM+F+I model. **C)** VDV-3 and VDV-5 were analysed as above with final alignment of 4082 nt and TIM2+F+G4 model. **D)** VDV-2 was analysed as above with final alignment of 8246 nt and GTR+F+I+G4 model. For A-D, branch supportsvwere estimated using Ultrafast bootstrap approximation (UFBoot; [3]) using 1000 replicates. Support values shown are SH-aLRT and UFBoot supports.

References:

1. Nguyen, L.-T.; Schmidt, H.A.; von Haeseler, A.; Minh, B.Q. Iq-tree: A fast and effective stochastic algorithm for estimating maximum-likelihood phylogenies. Mol Biol Evol 2014, 32, 268-274.
2. Kalyaanamoorthy, S.; Minh, B.Q.; Wong, T.K.F.; von Haeseler, A.; Jermiin, L.S. Modelfinder: Fast model selection for accurate phylogenetic estimates. Nat Methods 2017, 14.
3. Hoang, D.T.; Chernomor, O.; von Haeseler, A.; Minh, B.Q.; Vinh, L.S. Ufboot2: Improving the ultrafast bootstrap approximation. Mol Biol Evol 2017, 35, 518-522.

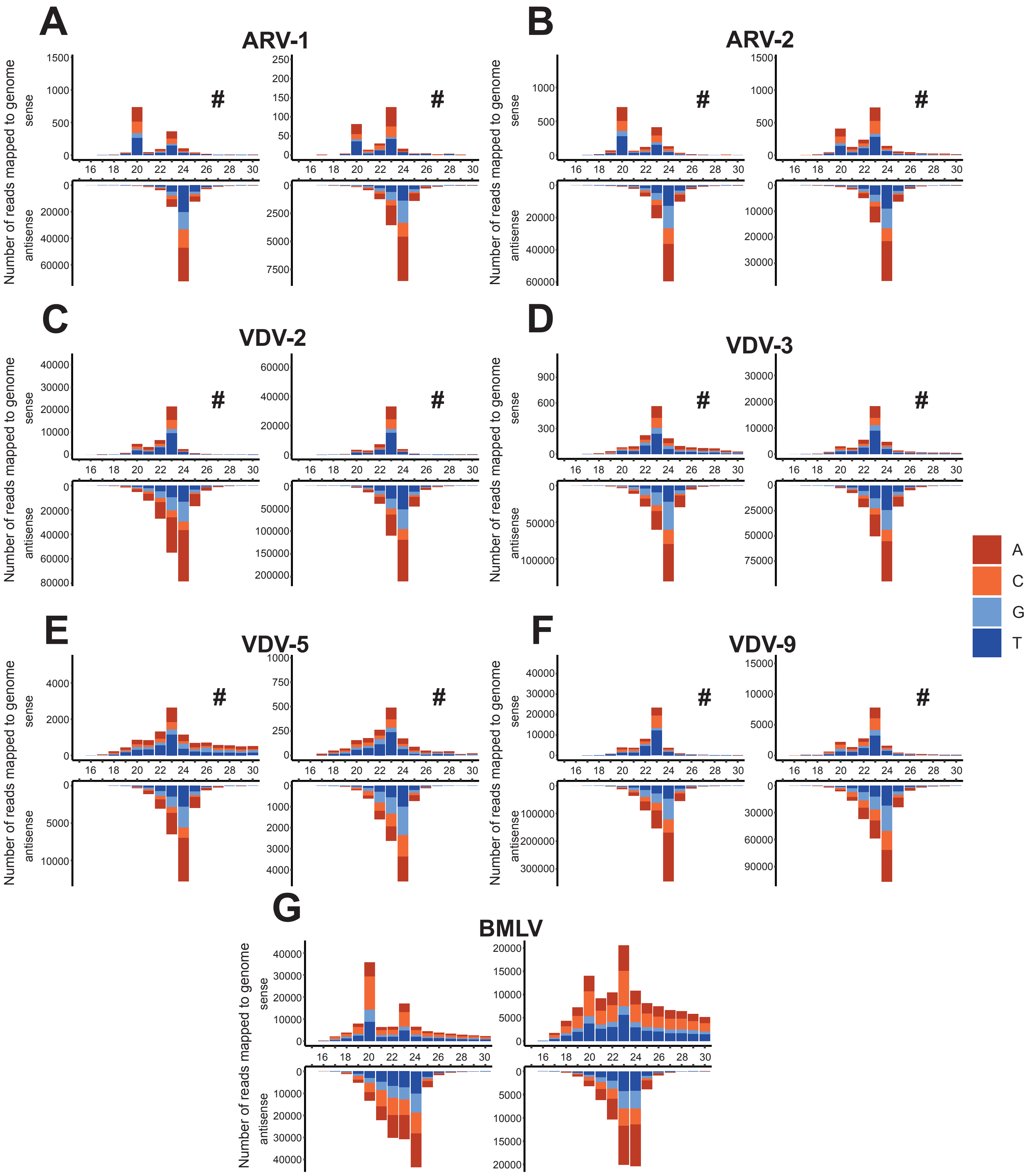

Figure S4. Additional samples showing representative size profiles of viral small RNA reads mapping to A) Apis rhabdovirus 1 (ARV-1: NE-5, SA-4); B) Apis rhabdovirus 2 (ARV-2: NE-2, SA-6p); C) Varroa destructor virus 2 (VDV-2: NE-8p, SA-5p); D) Varroa destructor virus 3 (VDV-3: CH-3, SA-5p); E) Varroa destructor virus 5 (VDV-5: NZ-8p, CH-1); F) Varroa destructor virus 9 (VDV-9: NZ-1, NE-1); G) Bee macula-like virus (BMLV: NE-6, NE-7p). # indicates sense Y-axis has been scaled to aid in visualising any size peaks.

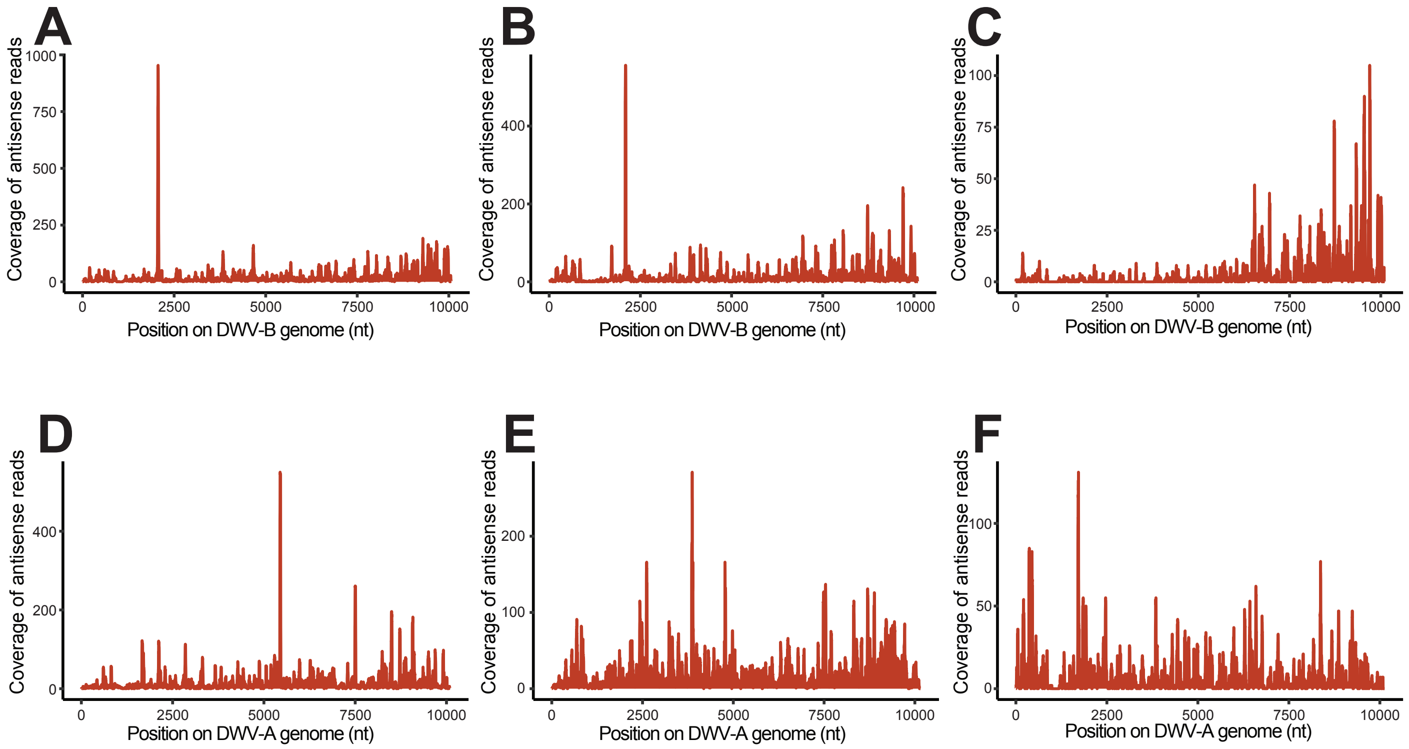

Figure S5. A) Coverage plots of antisense viral small RNA reads mapping to DWV-B (A-C) and DWV-A (D-E). Representative samples are shown for DWV-B (A: NE-1, B: NE-4, C: NE-8p) and DWV-A (D: NZ-5, E: US-2p, F: CH-2).
